# The lncRNA MIR2052HG regulates ERα levels and aromatase inhibitor resistance through LMTK3 by recruiting EGR1

**DOI:** 10.1186/s13058-019-1130-3

**Published:** 2019-04-03

**Authors:** Junmei Cairns, James N. Ingle, Krishna R. Kalari, Lois E. Shepherd, Michiaki Kubo, Matthew P. Goetz, Richard M. Weinshilboum, Liewei Wang

**Affiliations:** 1 Department of Molecular Pharmacology and Experimental Therapeutics, Mayo Clinic, Rochester, MN 55905 USA; 2 Division of Medical Oncology, Mayo Clinic, Rochester, MN 55905 USA; 3 Department of Health Sciences Research, Mayo Clinic, Rochester, MN 55905 USA; 4 NCIC Clinical Trials Group, Kingston, Ontario, K7L 3N6 Canada; 5 RIKEN Center for Integrative Medical Science, Yokohama City, 230-0045 Japan

**Keywords:** Lon noncoding RNA, MIR2052HG, EGR1, LMTK3, PKC, ERα, Aromatase inhibitor

## Abstract

**Background:**

Our previous genome-wide association study using the MA.27 aromatase inhibitors adjuvant trial identified SNPs in the long noncoding RNA *MIR2052HG* associated with breast cancer-free interval. MIR2052HG maintained ERα both by promoting AKT/FOXO3-mediated ESR1 transcription and by limiting ubiquitin-mediated ERα degradation. Our goal was to further elucidate MIR2052HG’s mechanism of action.

**Methods:**

RNA-binding protein immunoprecipitation assays were performed to demonstrate that the transcription factor, early growth response protein 1 (EGR1), worked together with MIR2052HG to regulate that lemur tyrosine kinase-3 (LMTK3) transcription in MCF7/AC1 and CAMA-1 cells. The location of EGR1 on the *LMTK3* gene locus was mapped using chromatin immunoprecipitation assays. The co-localization of MIR2052HG RNA and the *LMTK3* gene locus was determined using RNA-DNA dual fluorescent in situ hybridization. Single-nucleotide polymorphisms (SNP) effects were evaluated using a panel of human lymphoblastoid cell lines.

**Results:**

MIR2052HG depletion in breast cancer cells results in a decrease in LMTK3 expression and cell growth. Mechanistically, MIR2052HG interacts with EGR1 and facilitates its recruitment to the LMTK3 promoter. LMTK3 sustains ERα levels by reducing protein kinase C (PKC) activity, resulting in increased ESR1 transcription mediated through AKT/FOXO3 and reduced ERα degradation mediated by the PKC/MEK/ERK/RSK1 pathway. MIR2052HG regulated LMTK3 in a SNP- and aromatase inhibitor-dependent fashion: the variant SNP increased EGR1 binding to LMTK3 promoter in response to androstenedione, relative to wild-type genotype, a pattern that can be reversed by aromatase inhibitor treatment. Finally, LMTK3 overexpression abolished the effect of MIR2052HG on PKC activity and ERα levels.

**Conclusions:**

Our findings support a model in which the MIR2052HG regulates LMTK3 via EGR1, and LMTK3 regulates ERα stability via the PKC/MEK/ERK/RSK1 axis. These results reveal a direct role of MIR2052HG in LMTK3 regulation and raise the possibilities of targeting MIR2052HG or LMTK3 in ERα-positive breast cancer.

**Electronic supplementary material:**

The online version of this article (10.1186/s13058-019-1130-3) contains supplementary material, which is available to authorized users.

## Background

Estrogens have long been recognized to be important for stimulating the growth of estrogen receptor α (ERα)-positive breast cancer, a subtype that represents a large proportion of breast cancer patients. Estrogen action is mediated by ERα. Approximately 70% of breast cancers are ERα positive and rely on estrogen signaling to stimulate their growth and survival [1, 2]. Its presence in breast tumors is routinely used to predict response to endocrine therapies that target ERα, estrogen production or estrogen signaling. Aromatase inhibitors (AIs) suppress estrogen synthesis in postmenopausal women by targeting the aromatase enzyme, which converts precursor hormones to estrogens. The third-generation AIs (i.e., exemestane, anastrozole, and letrozole) have largely replaced tamoxifen as the preferred treatment for ERα-positive breast cancer in postmenopausal women with early-stage breast cancer because of their superior efficacy over tamoxifen [3, 4]. However, both de novo and acquired resistance to AIs can occur, resulting in relapse and disease progression. It is estimated that approximately 30% of ER-positive breast cancer receiving adjuvant AI treatment eventually develop resistance [5–7], while nearly all patients develop resistance in the metastatic setting. The mechanisms for endocrine therapy resistance are complex and one mechanism includes dysregulation of ERα expression, encoded by *ESR1* [8].

ERα is a member of the nuclear receptor superfamily of ligand-activated transcription factors [9], which regulates gene expression through direct binding to estrogen response elements (EREs) in promoters of estrogen-regulated genes and indirectly through recruitment to gene promoters by interaction with other transcription factors [10]. Previous studies have reported that ESR1 is upregulated during estrogen deprivation adaptation [11]. Overproduction of ERα leads to an enhanced response to low concentrations of estrogen, which is responsible for the acquisition of AI resistance or postmenopausal tumorigenesis [12, 13]. In these AI-resistant tumors, ERα is hypersensitive to low levels of estrogens [14] activated in a ligand-independent manner either by phosphorylation via kinases in the growth factor receptor signaling pathways or by acquired somatic *ESR1* mutations [15, 16]. ERα phosphorylation aids in regulating the transcriptional activity and turnover of ERα by proteasomal degradation. Of particular importance are Ser118 and Ser167, which locate within the activation function 1 region of the N-terminal domain of ERα and are regulated by multiple signaling pathways [17–20]. The phosphorylation at Ser118 can be mediated by mitogen-activated protein kinase (MAPK) activation and induces ERα activity [15, 21], whereas Ser167 can be phosphorylated by p90RSK [22, 23] and plays a role in lemur tyrosine kinase 3 (LMTK3)-mediated ERα stabilization [24, 25]. LMTK3 has been implicated in both de novo and acquired endocrine resistance in breast cancer [26]. The phosphorylation of ERα at S167 is positively associated with pMAPK and pp90RSK in breast cancer patients and a predictor of better prognosis in primary breast cancer with reduced relapse and better overall survival [27].

Our previous genome-wide association study (GWAS) used samples from the Canadian Cancer Trials Group MA.27, the largest AI breast cancer adjuvant endocrine therapy trial (4406 controls without recurrence of breast cancer and 252 cases with recurrence). In that study, we identified common single-nucleotide polymorphisms (SNPs) in a long noncoding (lnc) RNA, MIR2052HG, that were associated with breast cancer free interval (HR = 0.37, *p* = 2.15e−07) [28]. The variant SNPs (minor allele frequency [MAF] = 0.32 to 0.42) were associated with lower MIR2052HG and ERα expression in the presence of AIs, and two of the top SNPs, rs4476990 and rs3802201, were located in or near an ERE [28]. MIR2052HG appeared to affect ERα expression both by promoting AKT/FOXO3-mediated ESR1 transcription regulation and by limiting ubiquitin-mediated ERα degradation [28]. However, the underlying mechanisms by which MIR2052HG regulates ESR1 transcription and ERα degradation remain unknown.

Long noncoding RNA (lncRNAs) are transcripts with no protein-coding functions. Accumulating evidence suggests that lncRNAs play critical roles in regulating a wide range of cellular processes through affecting various aspects of protein, DNA, and RNA expression and interactions [29–31]. Several lncRNAs have been implicated in breast cancer. For example, UCA1 is an oncogene in breast cancer that can induce tamoxifen resistance [32]. LncRNA HOTAIR is positively correlated with tamoxifen resistance [33]. In the current study, we sought to further investigate the mechanism of MIR2052HG action in the regulation of ERα and AI resistance. We found that MIR2052HG directly interacts with the early growth response protein 1 (EGR1) protein to enhance LMTK3 transcription and thus sustained ESR1 expression and stabilized ERα protein.

## Methods

### Cell lines and chemical reagents

Human ER-positive breast cancer CAMA-1, HER-positive Au565, triple negative breast cancer (TNBC) MDA-MB-231, and human embryonic kidney cell line 293 T cell lines were obtained from American Type Culture Collection (ATCC). The identities of all cell lines were confirmed by the medical genome facility at Mayo Clinic Center (Rochester MN) using short tandem repeat profiling upon receipt. The breast cancer MCF7/AC1 cell line (stably transfected CYP19A1 gene) was provided by Dr. Angela Brodie (University of Maryland, Baltimore, MD). The cells were authenticated in 2015 by Genetica DNA Laboratories (Cincinnati, OH) using a StemElite ID system that uses short tandem repeat genotyping. CAMA-1cells were cultured in EMEM media (Eagle’s minimum essential medium) (ATCC) with 10% fetal bovine serum (FBS) (Corning) and MCF7/AC1 cells were cultured in IMEM (Improved MEM, no phenol red) (Life Technologies) with 10% heated inactive FBS in the incubator with 5% CO_2_ at 37°C. 293T cells were cultured in Dulbecco’s modified Eagle’s medium (Life Technologies) with 10% FBS, and Au565 cells were cultured in RPMI-1640 (Life Technologies) with 10% FBS in the incubator with 5% CO_2_ at 37 °C. MDA-MB-231 cells were cultured in L-15 medium with 10% FBS in the incubator without CO_2_. Five lymphoblastoid cell lines (LCLs) with MIR2052HG wild-type (WT) SNPs and five LCLs with MIR2025HG variant SNPs were selected. Before androstenedione treatment, ~ 2 × 10^7^ cells from each LCL were cultured in RPMI-1640 media containing 5% charcoal stripped FBS (Invitrogen) for 24 h, followed with RPMI-1640 medium without FBS for additional 24 h. Each LCL was plated into 12-well plates with RPMI-1640 medium containing 0.01, 0.1, 1, and 10 nM androstenedione. After 24 h treatment, increasing concentrations of anastrozole or exemestane were added. The anastrozole and exemestane concentrations ranged from 0.1, 1, 10, to 100 nM. After an additional 24 h, all LCLs were collected for further RNA extraction and qRT-PCR.

Anastrozole and exemestane (Selleckchem) were dissolved in DMSO (Sigma-Aldrich) as 100 mM stock. 4-Androstene-3, 17-dione (Steraloids Inc.) was dissolved in 100% ethanol. FLAG-ERα plasmid was provided by Thomas Spelsberg, Ph.D. (Mayo Clinic). FLAG-ERα S167A mutant was generated from the FLAG-ERα plasmid using QuickChange Site Directed Mutagenesis kit (Agilent). HA-Ub plasmid was provided by Zhenkun Lou, Ph.D. (Mayo Clinic). LMTK3 plasmid was purchased from Origene. Protein kinase C (PKC) inhibitor, Go 6983, was purchased from Sigma. Antibodies against GAPDH, AKT, p-AKT (Ser473), FOXO3, EGR1, EZH2, RSK1, pRSK1, ERK1/2, p-ERK1/2, MEK1/2, p-MEK1/2, and Phospho-(Ser) PKC Substrate Antibody were purchased from Cell Signaling. ERα and ERα-S167 antibodies were obtained from Abcam. LMTK3, BHLHE40, CTCF, EP300, HDAC6, POLR2A, REST, CREBBP, YY1, STAT1, and SNRNP70 antibodies were purchased from Santa Cruz. CHD2 antibody was from Thermo Fisher Scientific. Secondary HRP (horseradish peroxidase)-conjugated anti-rabbit IgG and anti-mouse IgG antibodies were from Cell Signaling.

### Antisense oligo knockdown and cDNA construct overexpression

Antisense oligonucleotides (ASOs) were used to knockdown and study the functions of MIR2052HG. Pools of two ASOs for MIR2052HG produced with locked nucleic acids modification of 5′ and 3′ ends (Exiqon) were validated previously [28]. Negative control ASO was obtained from Exiqon. Lipofectamine RNAi Max (Invitrogen) and OPTI-MEM (Life Technologies) were used for ASOs transfection. Knockdown efficiency was measured using TaqMan qRT-PCR. The sequences of ASOs were as follows: ASO1: 5′-GTTGATTAGATTTGG-3′; ASO2: 5′-ACAGTCCCGATCTTC-3′; negative control: 5′-AACACGTCTATACGC-3′. LMTK3 plasmids (Origene) were transfected into cells using Lipofectamine 2000 reagent (Thermo Fisher Scientific). Total RNA was extracted 48 h after transfection for RNA quantification. Whole cell lysates were collected 48 h after transfection for western blots.

### Quantitative real-time PCR assay (qRT-PCR)

QRT-PCR assays were performed for measuring gene expression using the TaqMan RNA-to-Ct 1-Step Kit (Life Technologies). RNA was extracted using the miRNeasy mini Kit (Qiagen). RNA was measured by NanoDrops300. The TaqMan primers for MIR2052HG, ESR1, and GAPDH were purchased from Life Technologies. Primers for EGR1 targeted genes and ESR1 targeted genes were purchased from IDT. QRT-PCR reactions were prepared following the manufacturer’s protocol. Samples were run using the StepOnePlus real-time PCR system (ABI).

### Western blotting

Cells were washed with cold PBS and were lysed in cold NETN buffer (100 mM NaCl, 20 mM Tris·HCl pH = 0.8, 0.5 mM EDTA, NP-40) with proteasome cocktail inhibitor and phosphatase inhibitor PhosSTOP EASY. Cell lysates were diluted with SDS loading buffer (SDS, glycerol, bromic acid, 1 M Tris·HCl) and boiled, centrifuged at 10,000 rpm, and stored at − 20 °C. Equal amounts of protein were subjected to electrophoresis in TGX SDS gels and were transferred to PVDF membranes. Membranes were blocked in TBS with 5% BSA and 0.1% Tween-20 and then incubated overnight at 4 °C with the indicated primary antibodies. Membranes were washed with TBS-T (TBS with 0.1% Tween-20) and then incubated with anti-mouse IgG or anti-rabbit IgG for 1 h at room temperature. All blots were visualized with the Supersignal WestPico or Supersignal WestDura chemiluminescent ECL kits (Thermo Fisher) and blue X-ray films or Gel Doc XR+ Gel documentation system (Biorad).

### RNA-seq analysis and normalization

RNA was prepared from MCF7/AC1 cells using the TRIzol extraction kit (Life Technologies). Genomic DNA was removed using the Ambion DNA-free kit. NuGEN Encore reagents were used for library preparation of total RNA samples. One microgram of total RNA input was used for each sample. The libraries were sequenced on an Illumina HiSeq 2000 sequencing system using 100-bp single-ended reads. After removing the poor-quality bases from FASTQ files for the whole transcriptome sequencing, paired-end reads were aligned by reads that were aligned to the human reference genome UCSC hg19 with Tophat 2.0.14 and the bowtie 2.2.6 aligner option. Transcript abundance was estimated using a count-based method with htseq-count.

### Cell proliferation and cell survival assays

Cells were seeded (2000 cells/100 μL/well) in a 96-well plate. The CyQUANT Direct Cell Proliferation Assay kit (Invitrogen) was used to determine the cell viability in six replicates. CyQUANT assays were performed to determine the cell viability every 2 days. Measurements were made using a microplate reader with excitation at 485 nm and emission detection at 530 nm. Each absorbance was normalized to the media control without any cells.

Cell survival assays were carried out in 96-well plates. Cells were seeded (5000 cells/100 μL/well) in a 96-well plate and treated with AIs for 72 h. CyQUANT assay was used to determine the cell viability in six replicates. Each absorbance was normalized to the media control without any cells.

### Colony forming assays

Cells transfected with MIR2052HG ASOs or LMTK3 plasmids were plated (800 ~ 1000 cells/well) in 6-well culture clusters in triplicates. Subsequently, the cells were cultured for up to 14 days at 37 °C, 5% CO_2_ to allow colony formation. Colonies were washed with cold PBS, fixed with 4% paraformaldehyde and stained by 0.05% crystal violet. Colonies (> 50 cells) were accounted with the ImageJ software (version 1.42q).

### Ubiquitination assays

Approximately1.5 μg of HA-ubiquintin plasmid and 1.5 μg of pcDNA 4.1-ERα plasmid with FLAG tag were co-transfected in HEK 293 T cells using lipofectamine 2000. Twenty-four hours later, cells were reversely transfected with the MIR2052HG ASO or negative control. Approximately 2 × 10^5^ ASOs transfected and control cells were subsequently seeded into each 60-mm dishes. After 64 h, MG132 was added at a final concentration of 10 μM for an additional 8 h. Cells were then collected for the ubiquitination assay. Specifically, these cells were washed in cold PBS with NEM (1:100) and lysed in 2% SDS lysis buffer [62.5 mM Tris·HCl pH = 6.8, 10% glycerol (*v*/v), SDS 2% (g/v)]. Immunoprecipitation assays were performed with the anti-FLAG antibody-conjugated gels (Sigma). After washing, the FLAG gels were dissolved in 2xSDS loading buffer and boiled. These samples were then subjected to western blotting using the anti-ubiquitin antibody and anti-FLAG antibody.

### PKC kinase assay

PepTag assay for nonradioactive detection of PKC Activity: MCF7/AC1 and CAMA-1 cells were transfected with indicated ASO or plasmids. Forty-eight hours later, cells were lysed and incubated with PKC reaction mixture (25 μl) according to the manufacturer’s (Promega) protocol at 30 °C for 30 min. The reactions were stopped by heating at 95 °C for 10 min. After adding 80% glycerol (1 μl), phosphorylated and nonphosphorylated PepTag peptides were separated by electrophoresis with 0.8% agarose gel. The negatively charged phosphorylated bands were excised using a razor blade, placed in a graduated microcentrifuge tube, and heated at 95 °C until the gel slice melted. The volume of the solution was adjusted to 250 μl with water. The hot agarose solution (125 μl) was added to a separate tube containing 75 μl of gel solubilization solution and 50 μl of glacial acetic acid. The absorbency was read at 570 nm. Using the absorbance, we calculated the number of units of kinase activity in each slice of agarose as per the manufacturer’s instructions (Promega). Assays were performed in triplicates.

Western Blot analysis of PKC activity was performed using lysates from MCF7/AC1 and CAMA-1 cells transfected with indicated ASO or plasmids. Phosphoserine PKC substrate proteins were detected by incubation overnight at 4 °C with anti-phosphoserine PKC substrate antibody.

### Chromatin immunoprecipitation (ChIP)

ChIP assays were performed using EpiTect ChIP OneDay kit (Qiagen). MCF7/AC1 and CAMA-1 cells were transfected with MIR2052HG ASO for 24 h. Cells were then subjected to ChIP assay as described by the manufacturer. LCLs were cultured in 5% charcoal stripped FBS for 24 h, followed with serum-free medium for additional 24 h. LCLs were then treated with 1 nM androstenedione, 1 nM androstenedione plus 100 nM anastrozole, and 1 nM androstenedione plus 100 nM exemestane for additional 24 h. Approximately 2 × 10^7^ LCLs per every sample (different SNP genotypes with androstenedione or androstenedione plus anastrozole or exemestane treatment groups) were collected for the ChIP assay. Equal amount of chromatin from each sample (~ 2 million cells each IP) and 1 μg control IgG or antibody against EGR1 were used. Q-PCR was carried out, and the result was normalized to input. All primers are listed in Additional file 1: Table S1.

### Fluorescent in situ hybridization (FISH)

The sequential protein staining and RNA detection were performed as previously described [34, 35]. Briefly, the cells were grown in chamber slides. LMTK3 staining was performed as usual until secondary antibody is labeled in the presence of RNase inhibitor. Slides were then dehydrated by serial treatment of ethanol with different concentrations. The Alexa Fluor 488-labeled RNA probe was obtained using the FISH Tag RNA Kit (Invitrogen). In the first step, in vitro transcription is used to enzymatically incorporate an amine-modified nucleotide into the probe template. The modified nucleotide is UTP having an NH2 group attached through a linker to the C5 position of the base. In the second step, dye labeling of the purified amine-modified RNA is achieved by incubation with amine-reactive dyes. These active ester compounds react with the primary amines incorporated into the probe template, covalently conjugating the dye to the modified nucleotide base. The purified probe is then ready for hybridization to the specimen slides at 37 °C overnight. Signal was then amplified using Tyramide Signal Amplification (TSA) kit (Life Technologies). LMTK3 DNA probe was produced using the FISH Tag DNA Kit (Invitrogen). In the first step, nick translation is used to enzymatically incorporate an amine-modified nucleotide into the probe template. The modified nucleotide is dUTP having an NH2 group attached through a linker to the C5 position of the base. In the second step, dye labeling of the purified amine-modified DNA is achieved by incubation with amine-reactive dyes. These active ester compounds react with the primary amines incorporated into the probe template, covalently conjugating the dye to the modified nucleotide base. The purified probe is then ready for hybridization to the specimen. For dual RNA-DNA-FISH, we used the protocol as previously described [35]. In brief, RNA-FISH was performed by using Nick-translated Alexa Fluor 488-labeled probe and followed by tyramide signal amplification kit as above. After RNA-FISH, the cells were treated by RNase A and denatured. Nick-translated BAC containing LMTK3 was labeled with Alexa Fluor 594 and used as probe. Images were obtained with the LSM 780 inverted confocal microscope runs on Zeiss’s Zen software package.

### RNA-binding protein immunoprecipitation

An RNA-binding protein immunoprecipitation (RIP) assay was performed using the Magna RIP kit (Millipore) according to the manufacturer’s instruction. Cell lysates from 50 million cells and 2–5 μg of control IgG or antibody against BHLHE40, CHD2, CTCF, EGR1, EP300, EZH2, HDAC6, POLR2A, REST, CREBBP, YY1, and STAT1 were used. We validated the RIP assay using the SNRNP70 antibody, which can bind to U1 snRNA. Specifically, cells were washed on the plates twice with 10 mL of PBS, scraped off from plate, and centrifuged at 1500 rpm for 5 min at 4 °C and discard the supernatant. Cell pellet was re-suspended in an equal pellet volume of complete RIP lysis buffer and then incubated on ice for 5 min. Dispense ~ 200 μL each of the lysate into nuclease-free microcentrifuge tubes and store at − 80 °C. Immunoprecipitations were performed using antibodies of interest and IgG control. Anti-SNRNP70 served as controls. Fifty micro liters of magnetic beads was washed and re-suspended in 100 μL of the RIP wash buffer. Two to approximately micrograms of the antibody of interest was added to each reaction and incubated with rotation for 30 min. The beads were then washed three times with RIP wash buffer. Nine hundred microliters of RIP immunoprecipitation buffer was then added to each tube. The RIP lysate were thawed and centrifuged at 14,000 rpm for 10 min at 4 °C, and 100 μL of the supernatant was added to each beads-antibody complex in RIP immunoprecipitation buffer. Ten microliters of the supernatant of RIP lysate was removed as “10% input” and stored at − 80 °C until starting RNA purification. The immunoprecipitations were incubated with rotating overnight at 4 °C, followed by six washes with 500 μL of cold RIP wash buffer. RNA purification was then performed. Each immunoprecipitate was re-suspended in 150 μL of proteinase K buffer. The input samples were thawed and 107 μL of RIP wash buffer, and 15 μL of 10% SDS and 18 μL of proteinase K were added to the tubes. All tubes were incubated at 55 °C for 30 min with shaking to digest the protein and then centrifuged briefly before being placed on the magnetic separator. The supernatant was then transferred into a new tube, together with 250 μL of RIP wash buffer. Four hundred microliters of phenol to chloroform to isoamyl alcohol was then added to each tube, followed by vortex for 15 s and centrifugation at 14000 rpm for 10 min to separate the phases. Three hundred microliters of the aqueous phase was carefully removed and placed in a new tube, together with 400 μL of chloroform. After vortex for 15 s and centrifugation at 14000 rpm for 10 min, the phases were separated. Three hundred microliters of the aqueous phase was carefully removed, and place it in a new tube. 50 μL of salt solution I, 15 μL of salt solution II, 5 μL of precipitate enhancer, and 850 μL of ethanol were added to each tube and kept at − 80 °C overnight to precipitate the RNA. The samples were then centrifuged at 14,000 rpm for 30 min at 4 °C. The pellets were washed once with 80% ethanol, air dried, and re-suspended in 10 to 20 μL of RNase-free water. The RNAs were then analyzed by qRT-PCR.

### Luciferase activity assay

Transcription activity of EGR1 was measured using the dual luciferase assay with the Cignal EGR1 Reporter Assay Kit (Qiagen). MIR2052HG knocked-down MCF7/AC1 and CAMA-1 cells were transfected with either EGR1 reporter (EGR1-responsive GFP reporter), negative control (GFP reporter construct with GFP expression controlled by a minimal promoter), or positive control (constitutively expressing GFP construct) constructs using the Lipofectamine 2000 transfection reagent. After 24 h of transfection, luciferase assay was performed using the Dual-Glo Luciferase Reporter Assay System (Promega) following the manufacturer’s protocol.

### Statistical analysis

For cell survival, cell proliferation, kinase activity, gene expression, and quantifications, data are represented as the mean ± SEM of three independent experiments. Statistical analyses were performed with Student’s *t* test. Statistical significance is represented in figures by *p < 0.05 and **p < 0.01.

## Results

### MIR2052HG regulates LMTK3 expression

We previously reported that MIR2052HG sustained ERα levels by promoting AKT/FOXO3-mediated upregulation of ESR1 transcript and by limiting proteasome-dependent degradation of ERα protein [28]. However, the mechanism involved in the regulation of MIR2052HG-mediated AKT activation and ERα ubiquitination remains unknown. Kinome screening previously identified LMTK3 as a potent ERα regulator, acting by decreasing the activity of protein kinase C (PKC) and the phosphorylation of AKT (Ser473), resulting in increased binding of FOXO3 to the *ESR1* promoter [24]. LMTK3 also protected ERα from proteasome-mediated degradation [24]. Given that the effects of LMTK3 on ERα were similar to our observations with MIR2052HG [28], we hypothesized that MIR2052HG might regulate LMTK3 to mediate ERα levels and, in turn, response to AIs.

**Fig. 1:**
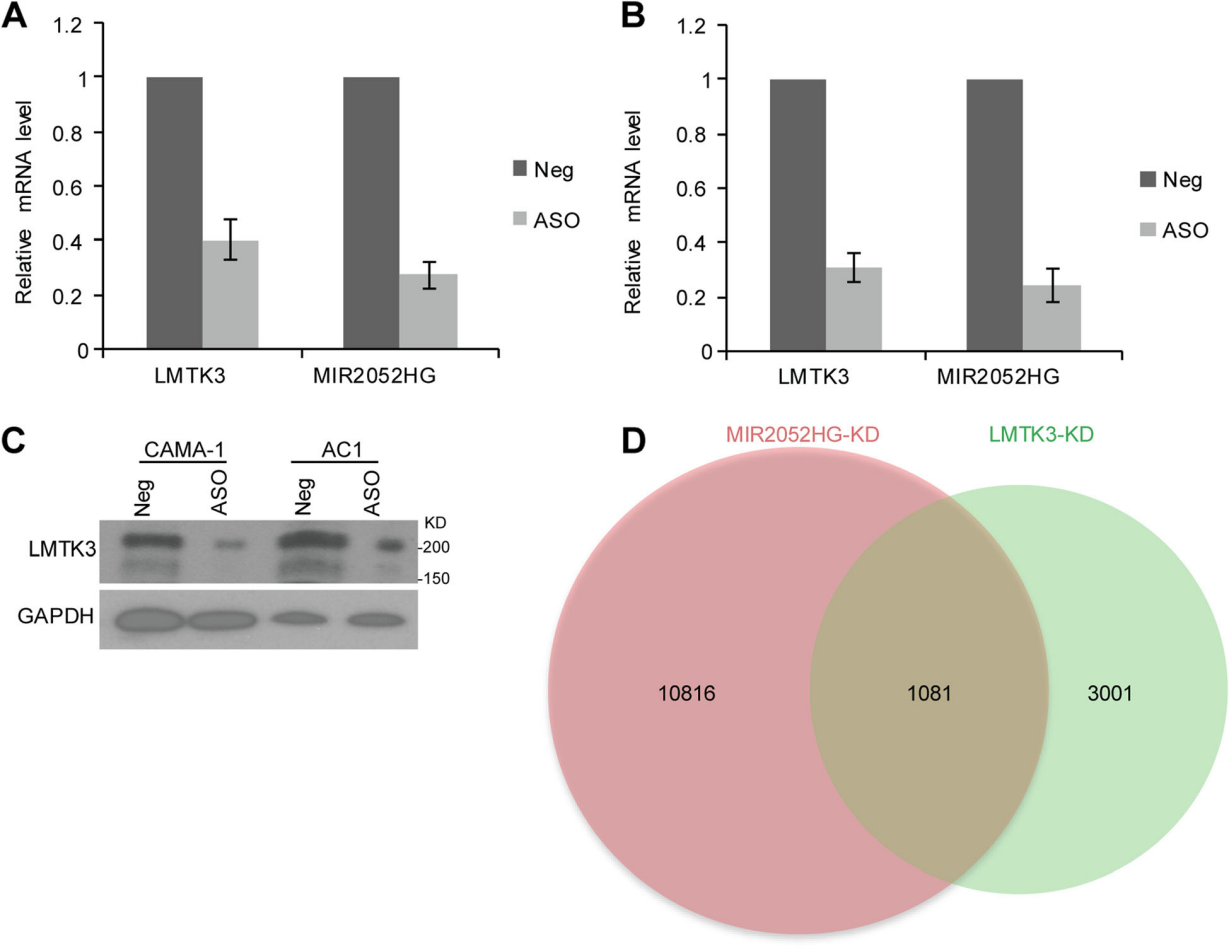
LMTK3 mRNA and protein expression in MIR2052HG knockdown cells. **a**–**b** Relative mRNA expression of LMTK3 after knockdown of MIR2052HG using pooled ASO in CAMA-1 (**a**) and MCF7/AC1 (**b**) cells. Error bars represent SEM; **p < 0.01 compared to baseline (negative control). **c** Western blots analysis of LMTK3 after knocking down MIR2052HG in CAMA-1 and MCF7/AC1 cell lines. **d** Venn diagram shows that genes affected by MIR2052HG knockdown from this study largely overlap with those published RNA-seq data after LMTK3-knockdown. MCF7/AC1 cells were transiently transfected with MIR2052HG ASO for 48 h. The RNA-seq data for LMTK3-knockdown were obtained from published data set [26]

Previous studies have demonstrated that lncRNAs can function in *trans* to regulate expression of protein-coding genes; therefore, we examined the possibility that MIR2052HG may facilitate AI resistance by regulating LMTK3 expression. Consistent with this hypothesis, we found that knockdown of MIR2052HG using a pooled ASO in human ER-positive CAMA-1 breast cancer cells resulted in a dramatic decrease of LMTK3 expression (Fig. 1a). A similar effect was also observed in an aromatase overexpressing cell line, MCF7/AC1 [36] (Fig. 1b). We also observed that the changes in mRNA levels were confirmed at the protein level by the western blot analysis (Fig. 1c), supporting the notion that MIR2052HG regulates LMTK3 expression. To determine whether LMTK3 is a major downstream target of MIR2052HG in regulating AI response, we first determined the transcriptome changes in MIR2052HG-knockdown MCF7/AC1 cells and collected published RNA-seq data after LMTK3-knockdown [26]. Analysis of the RNA-seq data indicated that the changes induced by MIR2052HG knockdown and LMTK3 knockdown showed a large number genes overlapped, especially almost 1/3 genes regulated by LMTK3 were also regulated by MIR2052HG (Fig. 1d, Additional file 2: Table S2). The common dysregulated genes in both knockdowns included cell cycle genes, oocyte maturation, and oocyte meiosis genes (Additional file 2: Table S2).

**Fig. 2:**
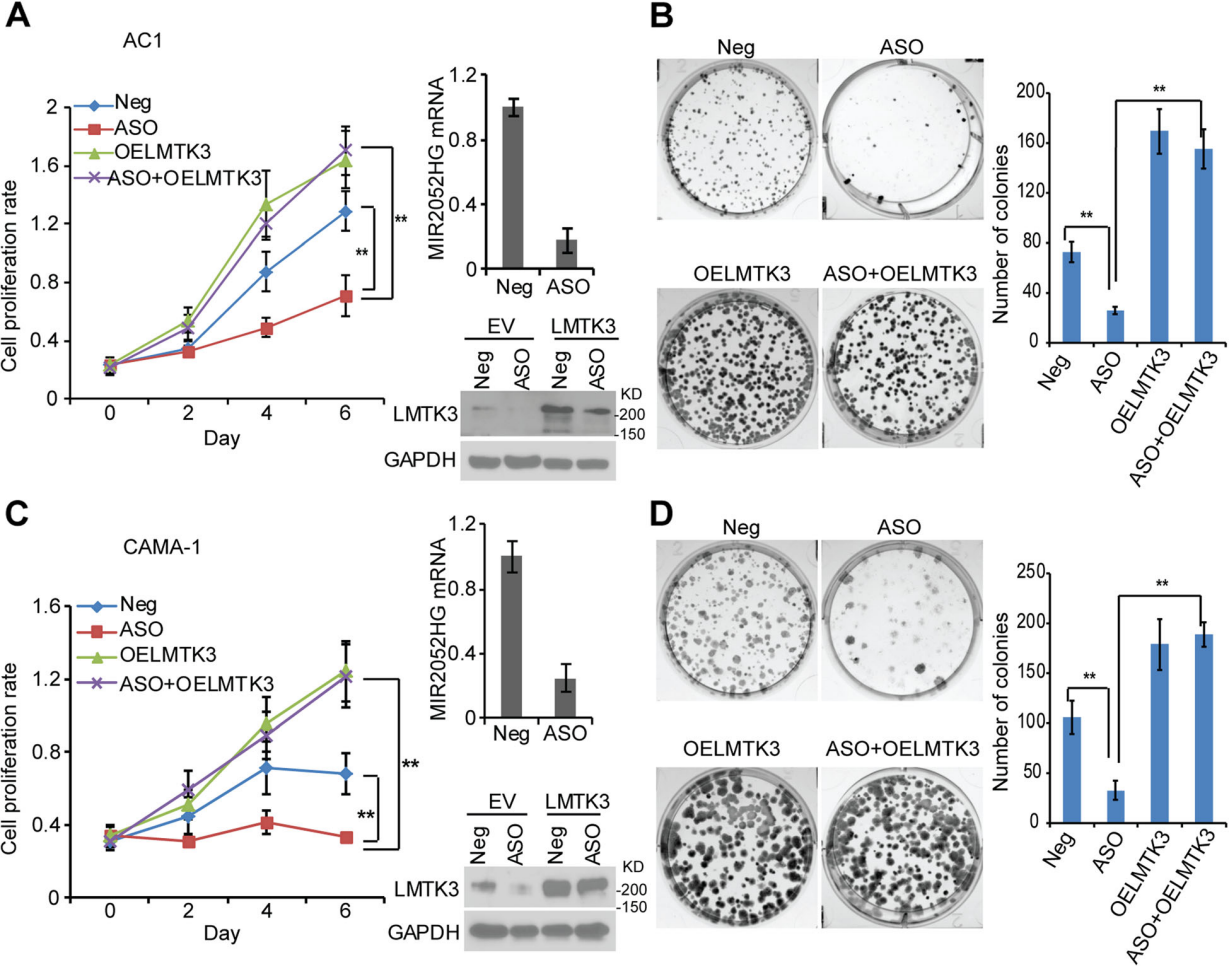
MIR2052HG regulates breast cancer cell growth through LMTK3- mediated signaling. **a**–**d** Overexpression of LMTK3 in MIR2052HG knocked-down MCF7/AC1 (**a**, **b**) and CAMA-1 (**c**, **d**) cells reversed the phenotypes of cell proliferation (**a**, **c**) and colony formation (**b**, **d**). Knockdown efficiency was determined by qRT-PCR. Overexpression of LMTK3 was determined by western blotting (**a**, **c**; right panel). Representative pictures of colony formation from three independent experiments are shown (**b**, **d**; left panel). The colony formation is quantified (**b**, **d**; right panel). Error bars represent SEM; **p < 0.01

To further define the relationship between MIR2052HG and LMTK3, we transfected LMTK3 overexpressing constructs into ERα-positive breast cancer cells with MIR2052HG-knockdown, followed by cell growth and colony forming assays. The cell proliferation and colony formation analysis demonstrated that the cell growth defect caused by downregulation of MIR2052HG could be successfully rescued by LMTK3 overexpression (Fig. 2a–d), indicating that LMTK3 is a major target that mediates the MIR2052HG regulation on cell growth in ER-positive breast cancer.

### LMTK3 mediates MIR2052HG-regulation of ESR1 transcription and ERα protein stability

**Fig. 3:**
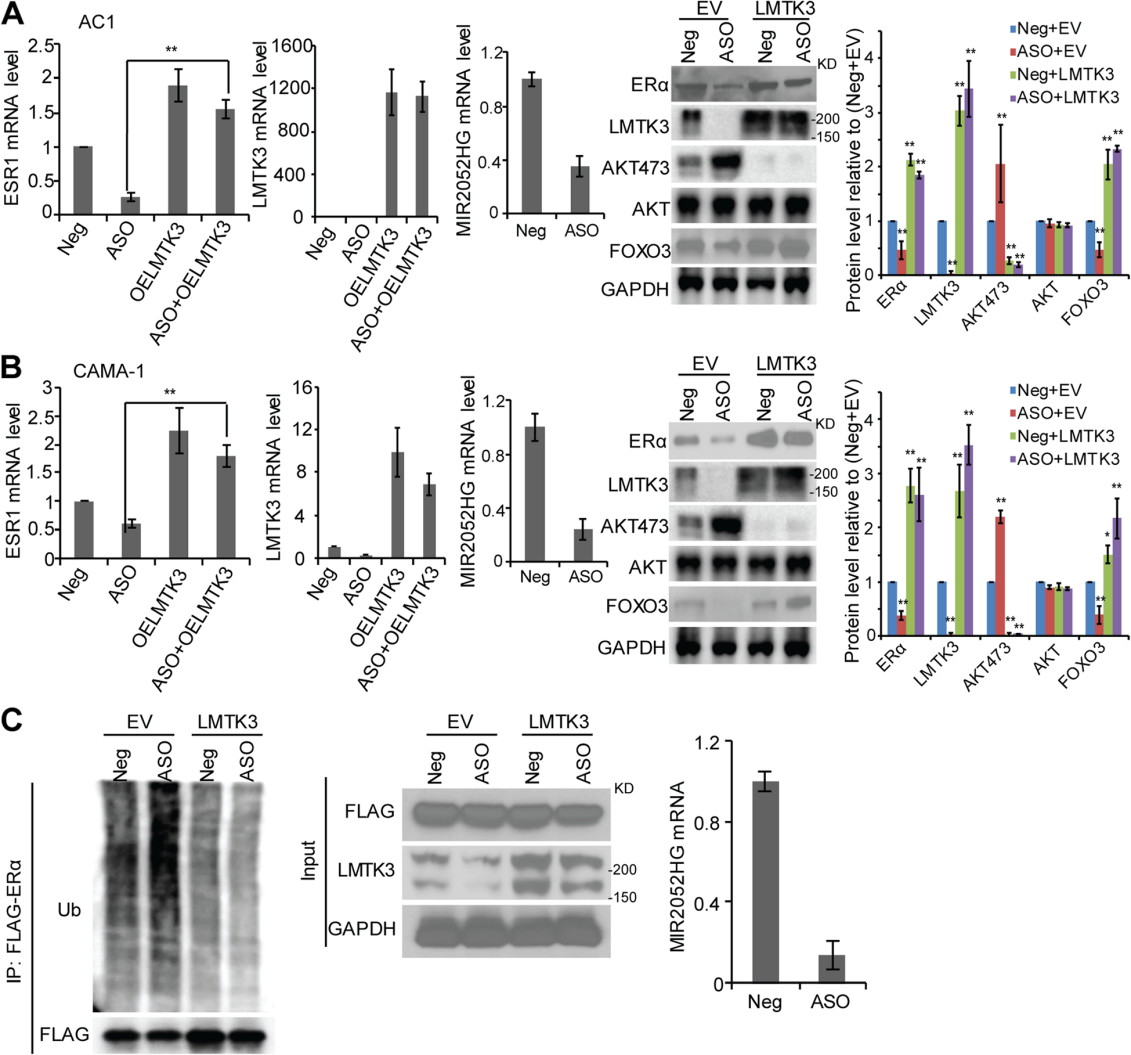
LMTK3-mediated MIR2052HG effect on regulation of ESR1 transcription and ERα protein stability. **a**–**b** Overexpression of LMTK3 in MIR2052HG knocked-down MCF7/AC1 (**a**) and CAMA-1 (**b**) cells reversed ERα protein and mRNA levels and, in turn, decreased AKT phosphorylation and FOXO3 level. Knockdown efficiency was determined by qRT-PCR. Overexpression of LMTK3 was determined by western blot analysis. Protein levels were quantified using ImageJ (right panel). Protein levels were normalized to GAPDH, and the normalized protein level in Neg+EV was set to 1. Error bars represent SEM. The significant difference between Neg+EV and all the other samples is indicated by **p < 0.01. **c** Overexpression of LMTK3 in MIR2052HG knocked-down cells reduced the ubiquitination of ERα. 293 T cells were transfected with HA-Ub plasmid and FLAG-ERα plasmid and then transfected with either the MIR2052HG ASOs or LMTK3 plasmid followed by MG132. ERα proteins were immunoprecipitated and analyzed by western blot analysis (left panel). Overexpression of LMTK3 was determined by western blot (middle panel). Knockdown efficiency was determined by qRT-PCR (right panel)

Previous studies demonstrated that MIR2052HG regulates ERα expression through transcription regulation of *ESR1* and ER protein degradation [24, 28]. However, the direct target of MIR2052HG in ERα regulation has not been fully elucidated. Therefore, we examined the role of LMTK3 in ERα regulation. Our previous report indicated that the effect of MIR2052HG on ESR1 transcript is through AKT/FOXO3 [24]. In MCF7/AC1 and CAMA-1 cells, downregulation of MIR2052HG reduced *ESR1* mRNA levels by promoting AKT-mediated downregulation of FOXO3 protein level, a transcription factor known to be involved in ESR1 transcription (Fig. 3a, b). LMTK3 overexpression rescued the downregulation of ERα mRNA induced by MIR2052HG silencing (Fig. 3a, b). LMTK3 overexpression resulted in a decrease in phosphorylated AKT (at Ser473) and an increase in FOXO3 protein level but not mRNA level (Fig. 3a, b, Additional file 3: Figure S1a). At the protein level, ERα protein was reduced by MIR2052HG knockdown, whereas LMTK3 overexpression stabilized ERα (Fig. 3a, b, right panel). Our previous results showed that MIR2052HG also regulated ERα stability by regulating its proteasome-dependent degradation process. Here, LMTK3 overexpression could increase protein level by decreasing ERα ubiquitination (Fig. 3c). Together, these data indicate that LMTK3, downstream of MIR2025HG, mediated MIR2052HG effect on the regulation of ESR1 transcription and ERα protein stability.

### MIR2052HG regulates ERα protein degradation through the LMTK3/PKC/MEK/ERK/RSK1 pathway

**Fig. 4:**
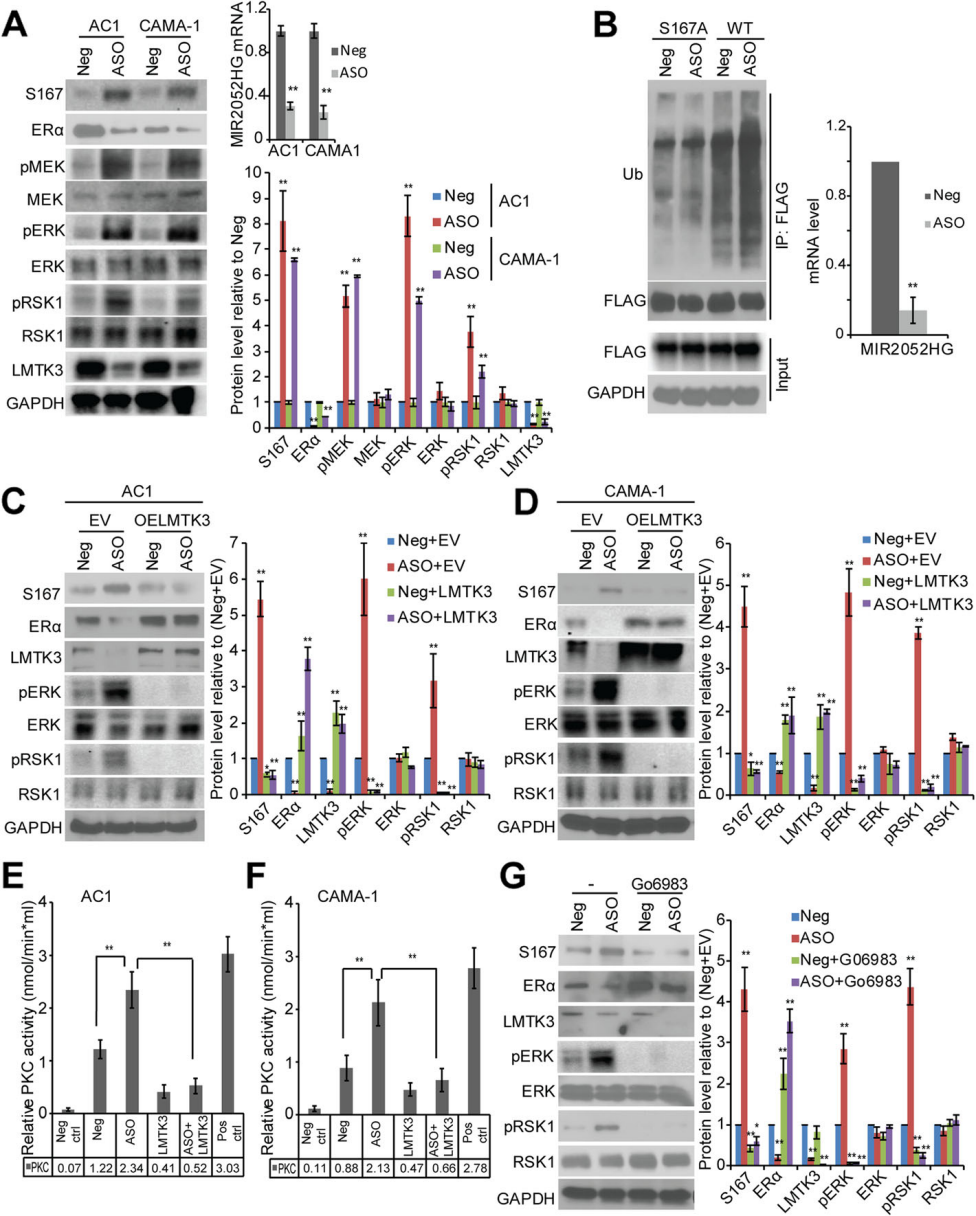
MIR2052HG regulates ERα protein stability through MEK/ERK/RSK1 pathway. **a** Knockdown of MIR2052HG increased phosphorylation of MEK, ERK, RSK1, as well as ERα S167 and decreased LMTK3 total level in MCF7/AC1 and CAMA-1 cells. Protein levels were quantified as described in Fig. 3. Error bars represent SEM. The significant difference between Neg and ASO is indicated by **p < 0.01. **b** Knockdown of MIR2052HG promoted the ubiquitination of wild-type ERα, but not ERα S167A mutant. 293 T cells were transfected with HA-Ub plasmid and FLAG-ERα or FLAG-ERα S167A plasmid and then transfected with either the MIR2052HG specific ASOs or the negative control ASO followed by MG132. Wild-type or S167A mutant ERα proteins were immunoprecipitated and analyzed by western blot. Knockdown efficiency in 293 T cells was determined by qRT-PCR. **c**–**d** Overexpression of LMTK3 in MIR2052HG knocked-down MCF7/AC1 (**c**) and CAMA-1 (**d**) cells reversed ERα protein levels and the phosphorylation of MEK, ERK, RSK1, and ERα S167. Overexpression of LMTK3 was determined by western blot analysis. Protein levels were quantified as described above. Error bars represent SEM. The significant difference between Neg+EV and all the other samples is indicated by: *p < 0.05, **p < 0.01. **e**–**f** PKC kinase assays examining the effect of MIR2052HG and LMTK3 on the catalytic activity of PKC in MCF7/AC1 (**e**) and CAMA-1 (**f**) cells. Error bars represent SEM of two independent experiments in triplicate; **p < 0.01. **g** Effects of MIR2052HG silencing on ERα protein levels in the presence of a PKC inhibitor (Go 6983). Protein levels were quantified as described above. Error bars represent SEM. The significant difference between Neg and all the other samples is indicated by *p < 0.05, **p < 0.01

Next, we investigated the mechanisms involved in MIR2052HG and LMTK3 regulation of ERα protein degradation. ERα phosphorylation, especially increased phosphorylation of ERα at Ser167, has been implicated in ERα proteasome-mediated degradation [24]. To explore the mechanism, we first determined the level of ERα pSer167 in MIR2052HG knockdown ERα-positive breast cancer cells. ERα pSer167 levels increased with MIR2052HG knockdown, despite decreased total ERα amounts (Fig. 4a). Furthermore, we observed that knocking down MIR2052HG increased wild-type (WT) ERα ubiquitination, but not the mutant ERα with serine 167 to alanine (S167A) (Fig. 4b), confirming the involvement of ERα Ser167 in ubiquitin-dependent and proteasome-mediated degradation. The phosphorylation of ERα at Ser167 is regulated by pp90 (RSK1) [22] which is activated by MAPK [37]. We thus hypothesized that MEK/ERK/p90RSK1 might be the signaling pathway that mediates ERα phosphorylation at Ser167 upon MIR2052HG knockdown. We first tested the effect of knockdown of MIR2052HG on MEK/ERK/p90RSK1 activity. As shown in Fig. 4a, in MCF7/AC1 and CAMA-1 cells transfected with MIR2052HG ASO, coinciding with increased ERα pSer167, pMEK, pERK, and pRSK1 levels were also increased, indicating an increased MEK/ERK/p90RSK1 activity. We then examined the role of LMTK3 in MIR2052HG-mediated regulation of MEK/ERK/p90RSK1 activity and found that LMTK3 overexpression abolished increased pMEK/pERK/p90RSK1 levels caused by MIR2052HG silencing, resulting in increased ERα protein (Fig. 4c, d) and its transcriptional activity (Additional file 3: Figure S1b). These data further imply that MIR2052HG regulates LMTK3 expression, which then influences MEK/ERK/p90RSK1 activity, regulating ERα protein levels.

As protein kinase C (PKC) has been implicated to play a role in ERα protein degradation [38] and AKT–FOXO3 regulation [39], and LMTK3 inhibits PKC activity [24], we examined the effects of MIR2052HG and LMTK3 on PKC. Downregulation of MIR2052HG increased PKC activity (Fig. 4e, f, and Additional file 3: Figure S1c), whereas overexpression of LMTK3 decreased PKC activity (Fig. 4e, f, and Additional file 3: Figure S1c). In addition, LMTK3 overexpression dramatically reduced PKC activity that was induced by MIR2052HG silencing (Fig. 4e, f, and Additional file 3: Figure S1c). Inhibition of PKC with the Go 6983 inhibitor reduced MEK/ERK/p90RSK1 activity and ERα pSer167, which in turn, partially rescued ERα levels (Fig. 4g), suggesting that MIR2052HG regulated ERα protein level through the axis of LMTK3/PKC/MEK/ERK/RSK1. Our findings also confirmed that MIR2052HG effects on AKT/FOXO3 activation and downstream ESR1 mRNA level were through the regulation of LMTK3/PKC pathway (Figs. 3 and 4).

### MIR2052HG contributes to LMTK3 transcription by facilitating EGR1 recruitment

**Fig. 5:**
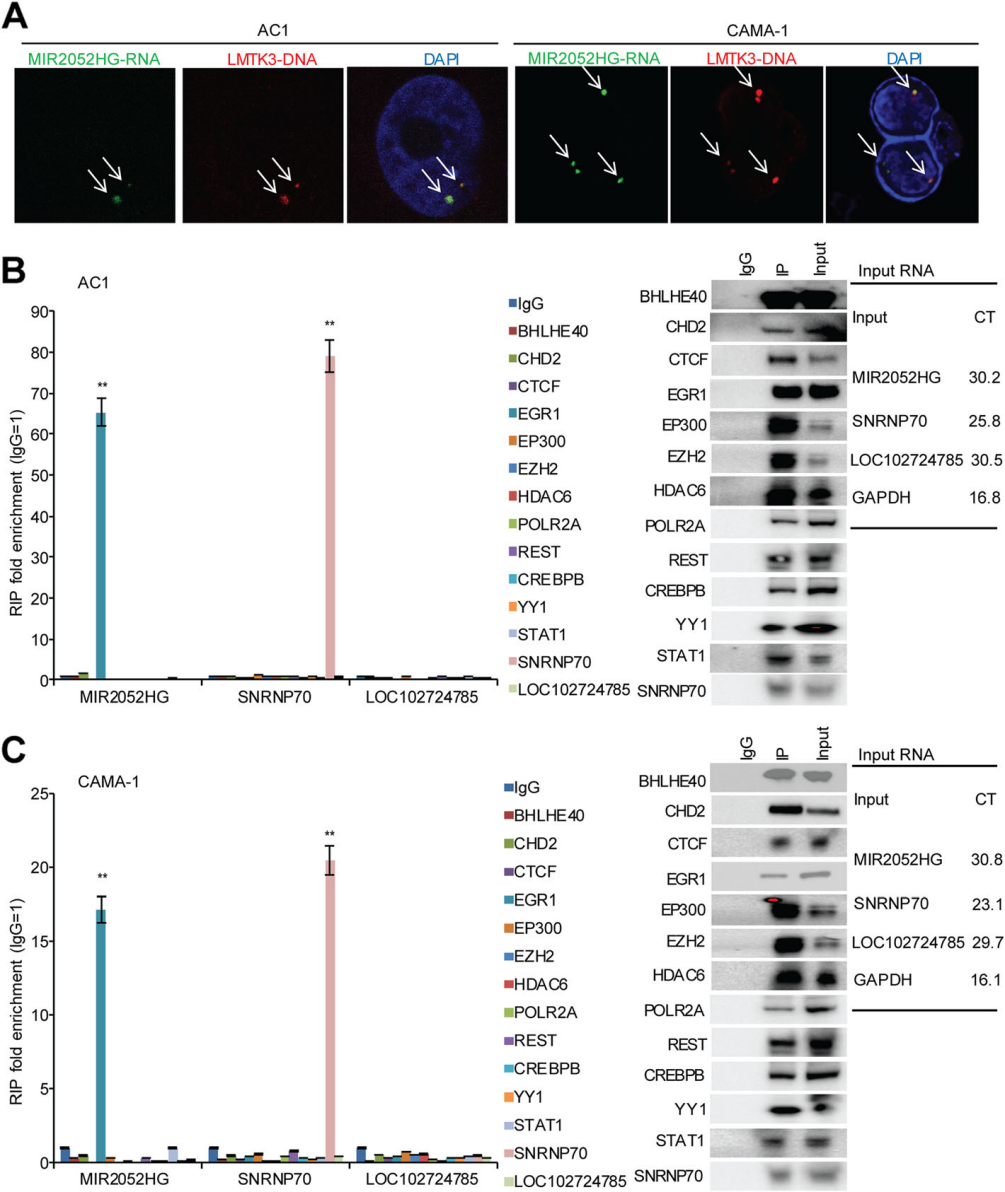
MIR2052HG regulates LMTK3 transcription by facilitating EGR1 recruitment to the LMTK3 promoter. **a** Dual RNA-DNA-FISH demonstrates that MIR2052HG transcripts (green signal) are localized onto the *LMTK3* gene locus (red signal) in MCF7/AC1 and CAMA-1 cells. **b**, **c** EGR1 antibody immunoprecipitates MIR2052HG, but not negative control lncRNA LOC102724785 in MCF7/AC1 (**b**) and CAMA-1 (**c**) cells. Error bars represent SEM of two independent experiments in triplicate; **p < 0.01

Next, we wanted to address how MIR2052HG regulates LMTK3 transcription. First, we examined the localization of the MIR2052HG RNA transcript. RNA-FISH demonstrated that MIR2052HG localized to a limited number of nuclear foci (one to two spots in most cases), suggesting that MIR2052HG had limited targets. We also checked the genomic location of the MIR2052HG transcript by RNA-DNA dual FISH, and the results showed that the MIR2052HG transcript was located at the *LMTK3* gene locus (Fig. 5a, Additional file 4: Figure S2). Taken together, these data suggest that MIR2052HG is likely involved in LMTK3 transcription.

LMTK3 expression could be activated by several transcription factors based on the ENCODE database, including BHLHE40, CHD2, CTCF, EGR1, EP300, EZH2, HDAC6, POLR2A, REST, CREBBP, YY1, and STAT1. Therefore, we asked whether any of these transcription factors, together with MIR2052HG might be involved in the regulation of MIR2052HG expression. Immunoprecipitation followed by qRT-PCR analysis demonstrated that MIR2052HG was significantly enriched in the EGR1 immunoprecipitates (Fig. 5b, c). The enrichment of MIR2052HG by the EGR1 antibody was specific, as the antibody did not pull down another lncRNA, LOC102724785 (Fig. 5b, c). Knockdown of MIR2052HG did not change LMTK3 expression in a HER2-positive Au565 and a TNBC MDA-MB-231 cell lines, and no significant enrichment of MIR2052HG by the EGR1 antibody was observed in AU565 and MDA-MB-231 cells (Additional file 5: Figure S3). These data suggest that MIR2052HG regulation of LMTK3 transcription involves EGR1 in ER-positive breast cancer.

**Fig. 6:**
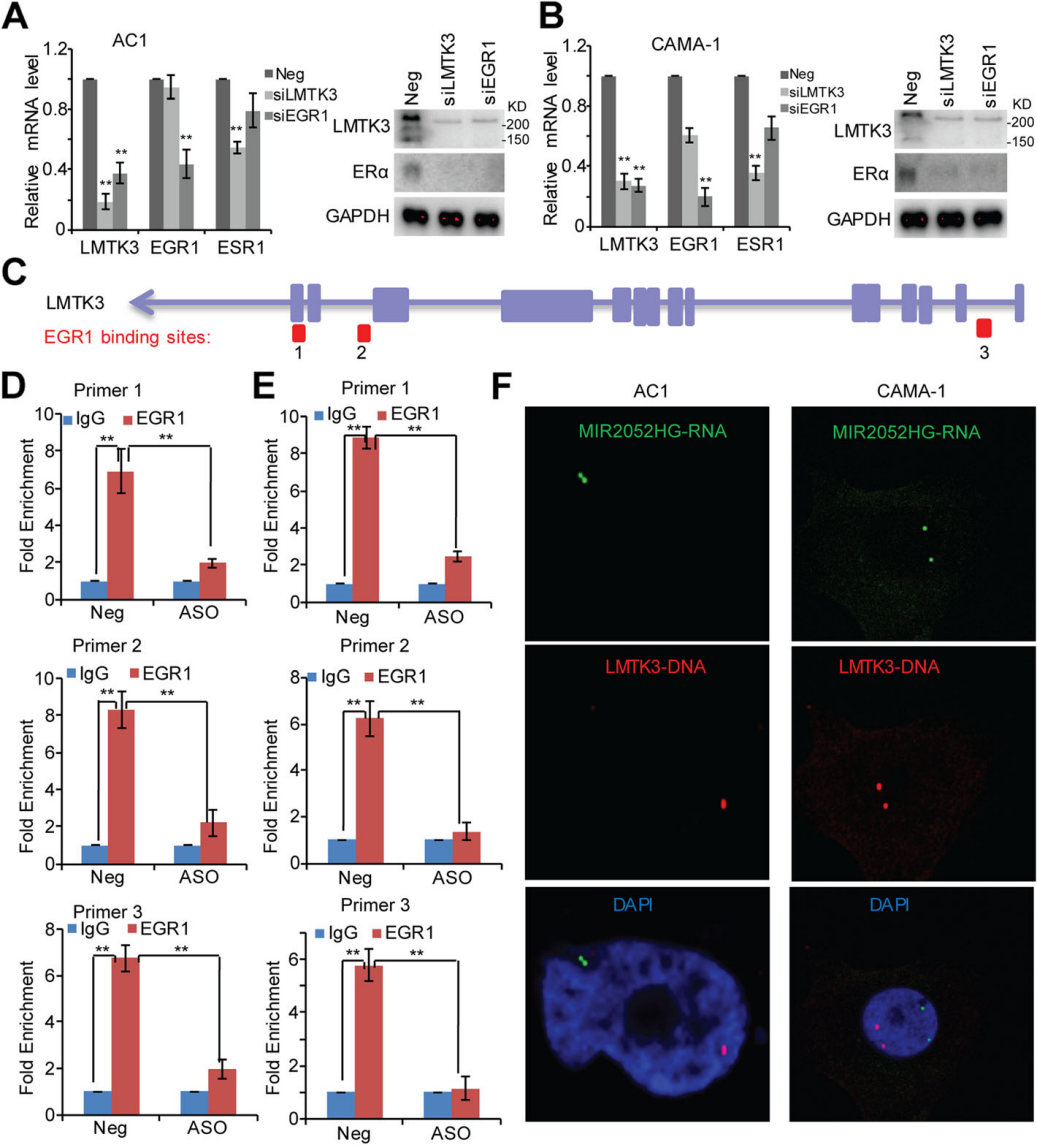
MIR2052HG regulates LMTK3 transcription by regulating EGR1 binding to its motif in *LMTK3* gene. **a**, **b** EGR1 regulates LMTK3 expression in MCF7/AC1 (**a**) and CAMA-1 (**b**) cells. **c** The EGR1 binding sites at the genomic location of the *LMTK3* gene locus are indicated in the diagram. **d**, **e** ChIP analysis demonstrates binding of EGR1 to *LMTK3* gene locus in MCF7/AC1 (**d**) and CAMA-1 (**e**) cells. IgG serves as a control. Error bars represent SEM of two independent experiments in triplicate; **p < 0.01. **f** Dual RNA-DNA-FISH demonstrates that MIR2052HG transcripts (green signal) fail to localize to the *LMTK3* gene locus (red signal) in EGR1 knockdown MCF7/AC1 and CAMA-1 cells

EGR1 was highly expressed in The Cancer Genome Atlas (TCGA) [40] ER-positive breast cancer patients (Additional file 6: Figure S4). We then confirmed EGR1 regulation of LMTK3 gene expression in the MCF7/AC1 and CAMA-1 cells. Knockdown of EGR1 reduced LMTK3 mRNA level (Fig. 6a, b). To examine whether binding of EGR1 to the *LMTK3* promoter requires MIR2052HG, we first mapped the binding locations of EGR1 on the *LMTK3* gene locus (Fig. 6c, chr19:48994366-48994811, chr19:48996320-48996559, chr19:49015095-49015334). ChIP assays demonstrated that EGR1 bound to all three binding sites (Fig. 6d, e). Importantly, knocking down MIR2052HG reduced the EGR1 binding to the *LMTK3* gene locus (Fig. 6d, e) without significant effect on the binding of EGR1 to other EGR1 targets (Additional file 7: Figure S5 and Additional file 8: Figure S6). Furthermore, MIR2052HG failed to locate in the *LMTK3* gene locus in EGR1 knockdown cells (Fig. 6f). Although EGR1 remained as a transcription factor for LMTK3 in HER2-positive and TNBC cells and knockdown LMTK3 inhibited cell growth (Additional file 9: Figure S7), knocking down MIR2052HG did not change the EGR1 binding to *LMTK3* gene locus (Additional file 9: Figure S7 c, d).

### AIs modulate LMTK3 expression in a *MIR2052HG* SNP-dependent manner

**Fig. 7:**
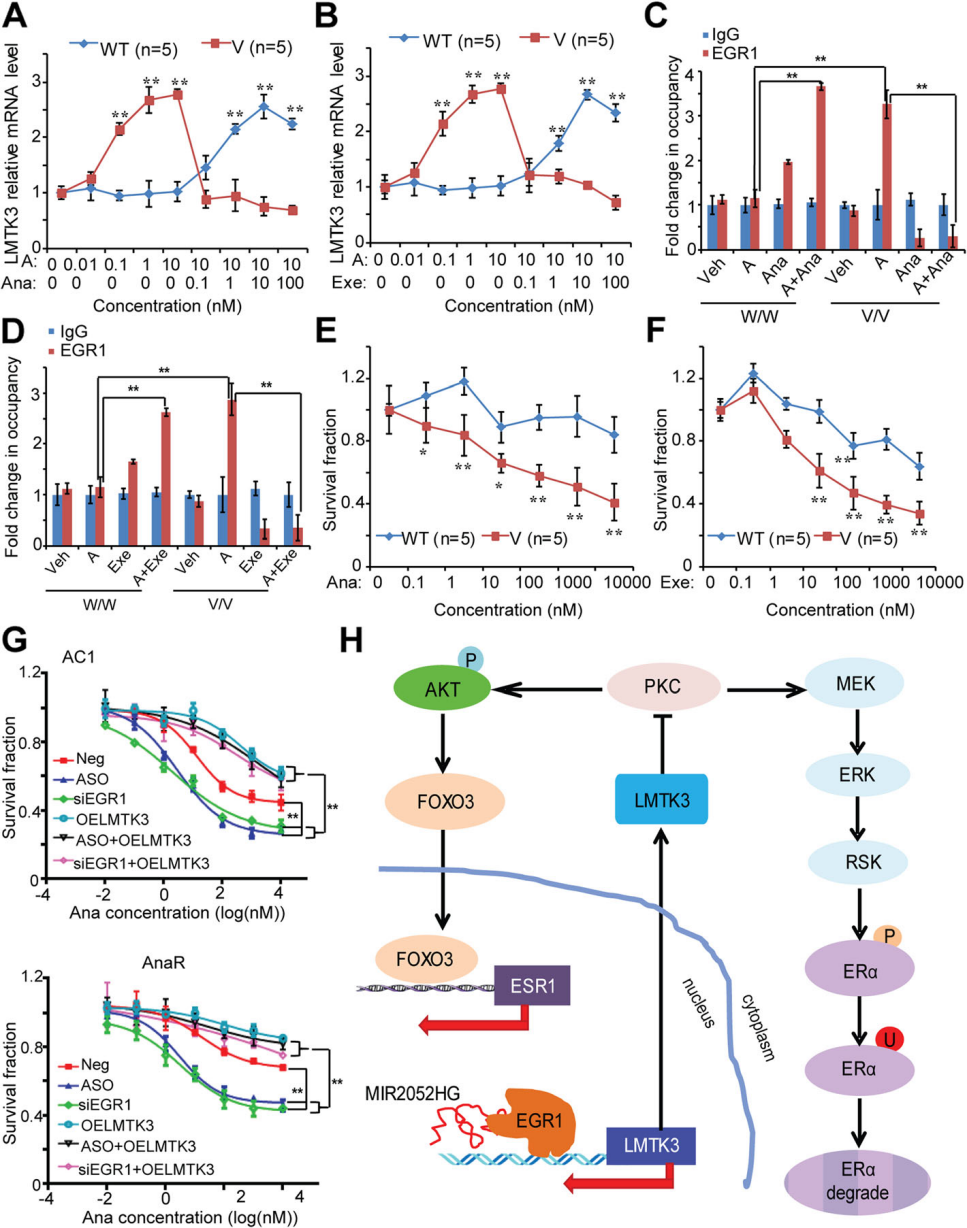
MIR2052HG-mediated SNP-dependent LMTK3 expression. **a**, **b** Androstenedione induction of MIR2052HG expression is associated with the expression of LMTK3. Five LCLs with either *MIR2052HG* WT or variant SNPs were exposed to treatments. LMTK3 expression levels were analyzed in each LCL and the averaged expression levels were shown for WT (*n* = 5) or V (*n* = 5) LCLs after exposure to androstenedione alone or with increasing concentrations of anastrozole (**a**) or exemestane (**b**). Error bars represent SEM. **p < 0.01. The concentrations for androstenedione (A), anastrozole (Ana), and exemestane (Exe) are indicated. **c**, **d** *MIR2052HG* SNPs determine androstenedione-dependent EGR1 binding to *LMTK3* gene locus. ChIP assay using pooled LCLs (*n* = 5) with known genotypes for MIR2052HG SNPs demonstrates binding of EGR1 to *LMTK3* gene locus after exposure to androstenedione alone or with anastrozole (**c**) or exemestane (**d**). Error bars represent SEM; **p < 0.01. **e**, **f** *MIR2052HG* SNP-dependent effect on AIs response. LCLs were treated with increasing dose of anastrozole (**e**) or exemestane (**f**) in the presence of 10 nM of androstenedione. Cell survival was analyzed 72 h after treatment for each LCL, and the averaged survival was shown for WT (*n* = 5) or V (*n* = 5) LCLs. Error bars represent SEM. *p < 0.05, **p < 0.01. **g** Effects of MIR2052HG-EGR1-LMTK3 on anastrozole response. Dose response of anastrozole in MCF7/AC1 and MCF7/AnaR cells. Cells were transfected with ASO or siEGR1, with or without overexpression of LMTK3 for 24 h, and then treated with anastrozole for 72 h. Error bars represent SEM. **p < 0.01. **h** Hypothetical model illustrated how MIR2052HG might regulate LMTK3 transcription. The red arrows indicate the transcription direction. The transcribed MIR2052HG interacts with EGR1 protein and brings EGR1 to the *LMTK3* locus. Together with other transcription machinery, binding of EGR1 to the LMTK3 promoter initiates transcription. LMTK3 protein inhibits the PKC, therefore downstream MAPK and AKT/FOXO3 pathways, leading to regulation of ERα degradation and ESR1 transcription

Our previous GWAS showed that *MIR2025HG* SNPs regulate its own gene expression as well as ERα expression in an estrogen or AI-dependent fashion [28]. Based on our findings showing MIR2025HG regulation of LMTK3, we then determined whether the expression of LMTK3 might be also SNP- and AI-dependent using the human LCLs system. This cell line model system, consisting of 300 individual LCLs for which we have extensive genomic and transcriptomic data, has shown repetitively to make it possible for us to study the relationship between common genetic variant and cellular phenotypes [28, 41, 42]. In the presence of androstenedione, LCLs with variant genotypes for both of the *MIR2052HG* SNPs, rs4476990 and rs3802201, showed dose-dependent increases in LMTK3 expression (Fig. 7a, b). However, addition of AI, either anastrozole (Fig. 7a) or exemestane (Fig. 7b) caused a “reversal” of the expression pattern with increased LMTK3 expression in LCLs with homozygous WT, but a marked decrease in LCLs homozygous for the variant genotypes. Of particular interest was the observation of a direct correlation between this pattern of expression for MIR2052HG and ERα [28] and that of LMTK3 (Fig. 7a, b). Since MIR2052HG regulated LMTK3 expression in a SNP- and AI-dependent fashion (Fig. 7a, b), we determined if EGR1 binding to the LMTK3 promoter region was also SNP- and AI-dependent. In the presence of androstenedione, cells homozygous for the variant SNP genotypes showed increased binding of EGR1 to the LMTK3 promoter (Fig. 7c, d) relative to WT in ChIP assays using the EGR1 antibody. Anastrozole and exemestane could reverse this effect (Fig. 7c, d).

We next sought to determine the functional consequences of the *MIR2025HG* SNP on response to AIs. LCLs homozygous for the variant SNP, which have low LMTK3 expression (Fig. 7a, b), were more sensitive to anastrozole and exemestane than homozygous WT LCLs (Fig. 7e, f). To assess the role of MIR2052HG-EGR1-LMTK3 axis in AI response, we next used anastrozole-sensitive MCF7/AC1 (because of its high expression of the AI target, CYP19A1), and anastrozole-resistant MCF7/AnaR [43] cell lines to determine the role of MIR2052HG-EGR1-LMTK3 axis in these two settings. In both lines, knockdown of MIR2052HG or EGR1 significantly increased anastrozole sensitivity compared to negative control, whereas overexpression of LMTK3 in MIR2052HG-knockdown or EGR1-knockdown cells resulted in decreased AI sensitivity (Fig. 7g). These results suggest that MIR2052HG facilitates EGR1 recruitment to the *LMTK3* promoter region in a SNP-dependent fashion to activate LMTK3 transcription, resulting in AI resistance.

## Discussions

Resistance to endocrine therapy represents a major challenge for ERα-positive breast cancer therapy. Therefore, the identification of biomarkers for endocrine response and understanding mechanisms of endocrine resistance should reveal possible strategies to overcome this problem. We have previously demonstrated that germline genetic variations in *MIR2052HG* were associated with breast cancer-free interval in the MA27 trial [28]. Downregulation of MIR2052HG reduced ERα-positive breast cancer cell growth. The variant SNPs were associated with increased MIR2052HG expression due to increased ERα binding to EREs [28]. Therefore, MIR2052HG plays an important role in regulating ERα and endocrine resistance [28]. Recently, LMTK3, a serine-threonine-tyrosine kinase, has gained attention in breast cancer with respect to its roles in pathogenesis and therapy resistance of breast cancer [24, 44, 45]. Using the TCGA data set, LMTK3 showed higher expression level in ER-positive breast cancer patients compared with normal breast and triple negative subtype (Additional file 10: Figure S8a, *p* = 6.5e−09 and *p* = 3.0e−11 respectively), and RNA expression levels were also independently associated with disease-free survival and overall survival (Additional file 10: Figure S8b). The fact that overexpression of LMTK3 significantly rescued the cell growth defect caused by MIR2052HG depletion suggests that LMTK3 is one of the downstream targets of MIR2052HG (Fig. 2). Furthermore, our data supported the notion that MIR2052HG *tran-*regulated LMTK3 transcription. MIR2052HG associated with EGR1 and facilitated its binding to the *LMTK3* gene promoter to activate LMTK3 expression (Figs. 5 and 6), which in turn, promoted ERα-positive breast cancer cell growth. As a direct target of MIR2052HG, LMTK3 regulated downstream PKC/AKT/FOXO3 and PKC/MAPK/RSK1/ERα signaling, therefore regulating ERα-positive breast cancer growth and AI response (Figs. 2, 3, and 4). Although MIR2052HG did not regulate LMTK3 expression in ERα-negative cells (Additional file 5: Figure S3), downregulation of LMTK3 inhibited ERα-negative cell proliferation (Additional file 9: Figure S7A, B), indicating LMTK3 may regulate other downstream pathways.

LncRNAs can play diverse roles in regulating gene expression as well as other cellular activities in breast cancer [46–48]. LncRNAs produce their cellular effects via several distinct mechanisms, including acting both *in cis* and *trans* [29, 30]. Here, we demonstrated that MIR2052HG exerted its oncogenic role by regulating LMTK3 expression. LMTK3 is significantly elevated in high-grade breast tumors and is associated with poor survival rates in different breast cancer cohorts [24, 26]. A prior study has shown that methylation is not a prevalent mechanism in the control of LMTK3 expression in breast cancer, and several somatic mutations in LMTK3 have been associated with overall survival [24]. However, we did not find any germline variations in *LMTK3* associated with breast cancer recurrence in our MA.27 cohort, suggesting a LMTK3 upstream regulator such as MIR2052HG might be the driving factor influencing this clinical phenotype. We found that MIR2052HG was induced by hormone or AIs, and it was required for the LMTK3-mediated phenotypes, including cell growth in response to AIs (Fig. 7). Current research into the potential role of LMTK3 as a therapeutic target is underway [49, 50]. At mechanistic level, we found that MIR2052HG positively regulated ERα at both mRNA and protein levels via LMTK3 to maintain the cancer cell growth. LMTK3 mediated the effect of MIR2052HG on AI response via ERα transcription through the LMTK3/PKC/AKT/FOXO3 signaling and protein levels via the LMTK3/PKC/MAPK pathway (Figs. 3 and 4). We also found a positive correlation between the expressions of LMTK3 and ESR1 (Additional file 10: Figure S8c and d) in the METABRIC and TCGA set data sample set [40], as well as in our LCLs model (*p* = 3.45e−04, rho = 0.212). Due to the low expression levels of MIR2052HG in some of the patient samples (Additional file 6: Figure S4), we did not find strong correlation between the expressions of MIR2052HG and LMTK3, and the correlations between MIR2052HG RNA expression levels and disease-free survival or overall survival in TCGA cBioPortal are not available.

EGR1 is an immediate-early gene induced by estrogen, growth factors, or stress signals [51]. The EGR1 protein binds to a specific GC-rich sequence in the promoter region of many genes to regulate the expression of these target genes including growth factors and cytokines. The mechanisms by which EGR1 activates downstream target genes appears to be cell-context dependent [52–54]. Although the DNA-binding domain of EGR1 is capable of binding to DNA through the GC-rich consensus sequence GCG (G/T) GGGCG, EGR1 can act as either an activator or a repressor of transcription through mechanisms that depend on interactions with distinct cofactors, and thus many partners, including DNA-binding proteins, have been reported to form complexes with EGR1 to activate EGR1 target gene expression [55, 56]. In our study, we demonstrated that the association of MIR2052HG with EGR1 facilitated EGR1 binding to the *LMTK3* gene locus (Figs. 5 and 6). Based on the current data, we propose a hypothetical model that may explain how MIR2052HG contributes to LMTK3 activation and AI resistance (Fig. 7h). In the model, we showed that MIR2052HG facilitated the recruitment of EGR1 to the *LMTK3* gene through its interaction with EGR1and activated LMTK3 transcription. This process might also involve other transcription cofactors. It is possible that other proteins are also required for the binding of MIR2052HG to EGR1, since some RNA-binding proteins have been shown to be able to regulate EGR1 [57]. Although our data showed that EGR1 binds to three binding sites in *LMTK3* gene locus and MIR2052HG affects all three sites binding activity (Fig. 6d, e), this study did not address the difference in gene regulation among the three EGR1 binding sites. One potential explanation could be the existence of homotypic clusters, that is, many adjacent transcription factor binding sites for the same transcription factor. Homotypic clusters might influence gene regulation through cooperativity or no cooperativity mechanisms [58]. Future studies may also explore whether the three binding sites involve in transcribing different LMTK3 variants. Nevertheless, RNA-mediated EGR1 targeting represents one mechanism by which EGR1 is recruited to its targets.

## Conclusions

Our findings support a model in which the protective MIR2052HG variant genotype regulates LMTK3 expression by enhancing the recruitment of ERG1 to the *LMTK3* promoter region, activating its transcription. At the mechanistic level, LMTK3 regulates ERα stability via the PKC/MEK/ERK/RSK1 axis and ERα transcription through PKC/AKT/FOXO3 pathway. This regulation may explain the effect of the MIR2052HG variant genotype on cell proliferation and response to AIs in MA.27. These findings provide new insight into the mechanism of action of MIR2052HG and suggest that LMTK3 may be a new therapeutic target in ERα-positive breast cancer patients, especially those who might not respond to AIs.

## Additional files

Additional file 1: **Table S1.** Primers for LMTK3 CHIP assay. (XLSX 9 kb)

Additional file 2: **Table S2.** RNA-seq results after MIR2052HG and LMTK3 knockdown. Overlapped genes between knockdown MIR2052HG and knockdown LMTK3 are highlighted. Pathway analysis of common dysregulated genes in both MIR2052HG and knockdown LMTK3 knockdowns. (XLSX 1523 kb)

Additional file 3: **Figure S1.** LMTK3 mediates MIR2052HG- regulation of ERα. a Overexpression of LMTK3 in MIR2052HG knocked-down MCF7/AC1 and CAMA-1 cells did not change AKT and FOXO3 mRNA levels. b Expression profiles of ER target genes in MCF7/AC1 and CAMA-1 cells. Cells were transfected with ASO and LMTK3 plasmid. RNA was prepared 24 h following transfection. c Effects of MIR2052HG and LMTK3 on the ability of PKC to phosphorylate its substrates. (TIF 1963 kb)

Additional file 4: **Figure S2.** *LMTK3* DNA FISH probe map with two options for BACs that cover *LMTK3* gene region which were 166 kb and 215 kb. (TIF 3156 kb)

Additional file 5: **Figure S3.** Knockdown of MIR2052HG does not affect LMTK3 expression and proliferation of HER2+ and TNBC cells. a–b Cell proliferation of HER+ Au565 (a) and TNBC MDA-MB-231 (b) cells after knocking down MIR2052HG. LMTK3 gene expression and MIR2052HG knockdown efficiency was determined by qRT-PCR. c–d EGR1 antibody failed to immunoprecipitate MIR2052HG in Au565 (c) and MDA-MB-231 (d) cells. Error bars represent SEM of two independent experiments in triplicate. (TIF 1019 kb)

Additional file 6: **Figure S4.** MIR2052HG and EGR1 expression in TCGA ER-positive breast cancer patients. (TIF 1311 kb)

Additional file 7: **Figure S5.** Knockdown of MIR2052HG specifically reduces binding of EGR1 to the *LMTK3* promoter, but not the other EGR1 targets. a–b Relative mRNA expression of EGR1 targeted genes after knockdown of EGR1 in MCF7/AC1 (a) and CAMA-1 (b) cells. Error bars represent SEM; *p < 0.05, **p < 0.01. c–d Relative mRNA expression of EGR1 targeted genes after knockdown of MIR2052HG in MCF7/AC1 (c) and CAMA-1 (d) cells. Error bars represent SEM; *p < 0.05, Non-significant (NS): *p* > 0.05. (TIF 1454 kb)

Additional file 8: **Figure S6.** MIR2052HG has no significant effect on other EGR1 targeted genes. a EGR1 reporter assay in MIR2052HG knocked-down MCF7/AC1 and CAMA1 cells. b ChIP analysis demonstrates binding of EGR1 to additional EGR1 targeted genes and knockdown of MIR2052HG has no impact on the binding. IgG serves as a control. Error bars represent SEM; Non-significant (NS): *p* > 0.05. (TIF 848 kb)

Additional file 9: **Figure S7.** EGR1, but not MIR2052HG, regulates LMTK3 expression in HER2+ and TNBC cells. a–b Cell proliferation of HER+ Au565 (a) and TNBC MDA-MB-231 (b) cells after knocking down LMTK3. LMTK3 gene expression and EGR1 knockdown efficiency was determined by qRT-PCR. c–d ChIP analysis demonstrates the binding of EGR1 to the promoter region of the *LMTK3* gene locus in AU565 (c) and MDA-MB-231 (d) cells. However, knockdown of MIR2052HG did not change the binding. IgG serves as a control. Error bars represent SEM of three independent experiments in triplicate; **p < 0.01, Non-significant (NS): *p* > 0.05. (TIF 1117 kb)

Additional file 10: **Figure S8.** Correlations of LMTK3 expression with ESR1. a LMTK3 expression in TCGA breast cancer patients. There are significant differences in the mean expression of LMTK3 among the four groups (HER2, ER+, TN, and Normal) using Kruskal-Wallis test (*p* < 2.2e−16). Pairwise comparison Wilcoxon test was also performed to determine the LMTK3 expression difference between the subtypes. Using Bonferroni correction for multiple testing, pairwise comparison showed: LMTK3 level in TN subtype is different from HER2 (*p* = 2.4e−08) and ER+ (*p* = 3.0e−11) but not significantly different from Normal (*p* = 0.17); LMTK3 in HER2 subtype is different from Normal (*p* = 1.5e−07) but not from ER positive (*p* = 0.892); while ER+ is significantly different from Normal (*p* = 6.5e−09). b Kaplan-Meier plots demonstrated the associations between LMTK3 expression level and overall survival (*p* = 3.927e−5) as well as disease-free survival (*p* = 9.587e−5) in TCGA ER-positive breast cancer patients. c Correlations of LMTK3 expression with ESR1 in 2509 METABRIC breast cancer patients. d Correlations of LMTK3 expression with ESR1 in TCGA breast cancer patients. (TIF 2271 kb)

## Abbreviations

AI: Aromatase inhibitor
ASO: Antisense oligonucleotides
ChIP: Chromatin immunoprecipitation
EGR1: Early growth response protein 1
ERE: Estrogen response element
ERα: Estrogen receptor α
FISH: Fluorescent in situ hybridization
GWAS: Genome-wide association study
LCL: Lymphoblastoid cell line
LMTK3: Lemur tyrosine kinase-3
LncRNA: Long noncoding RNA
PKC: Protein kinase C
qRT-PCR: Quantitative real-time PCR assay
RIP: RNA-binding protein immunoprecipitation
SNP: Single-nucleotide polymorphisms
TCGA: The Cancer Genome Atlas
WT: Wild type
