# Supplementary figures and images for: The lncRNA MIR2052HG regulates ERα levels and aromatase inhibitor resistance through LMTK3 by recruiting EGR1
Source: Breast Cancer Res. 2019 Apr 3;21:47. doi: 10.1186/s13058-019-1130-3 (PMC6448248; doi:10.1186/s13058-019-1130-3)

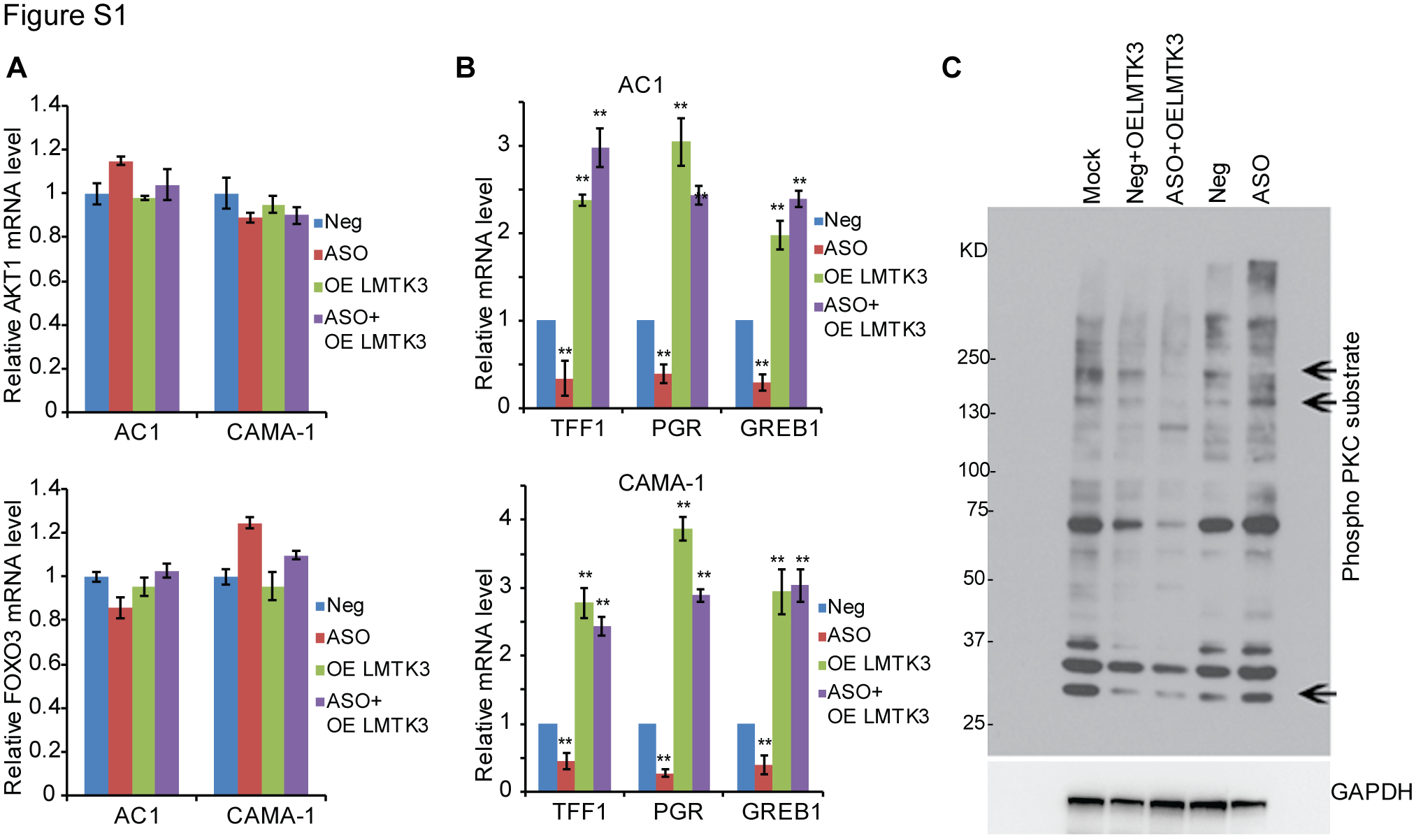

Supplement: Supplementary file 3 — Figure S1. LMTK3 mediates MIR2052HG- regulation of ERα. a Overexpression of LMTK3 in MIR2052HG knocked-down MCF7/AC1 and CAMA-1 cells did not change AKT and FOXO3 mRNA levels. b Expression profiles of ER target genes in MCF7/AC1 and CAMA-1 cells. Cells were transfected with ASO and LMTK3 plasmid. RNA was prepared 24 h following transfection. c Effects of MIR2052HG and LMTK3 on the ability of PKC to phosphorylate its substrates. (TIF 1963 kb) [file 13058_2019_1130_MOESM3_ESM.tif]

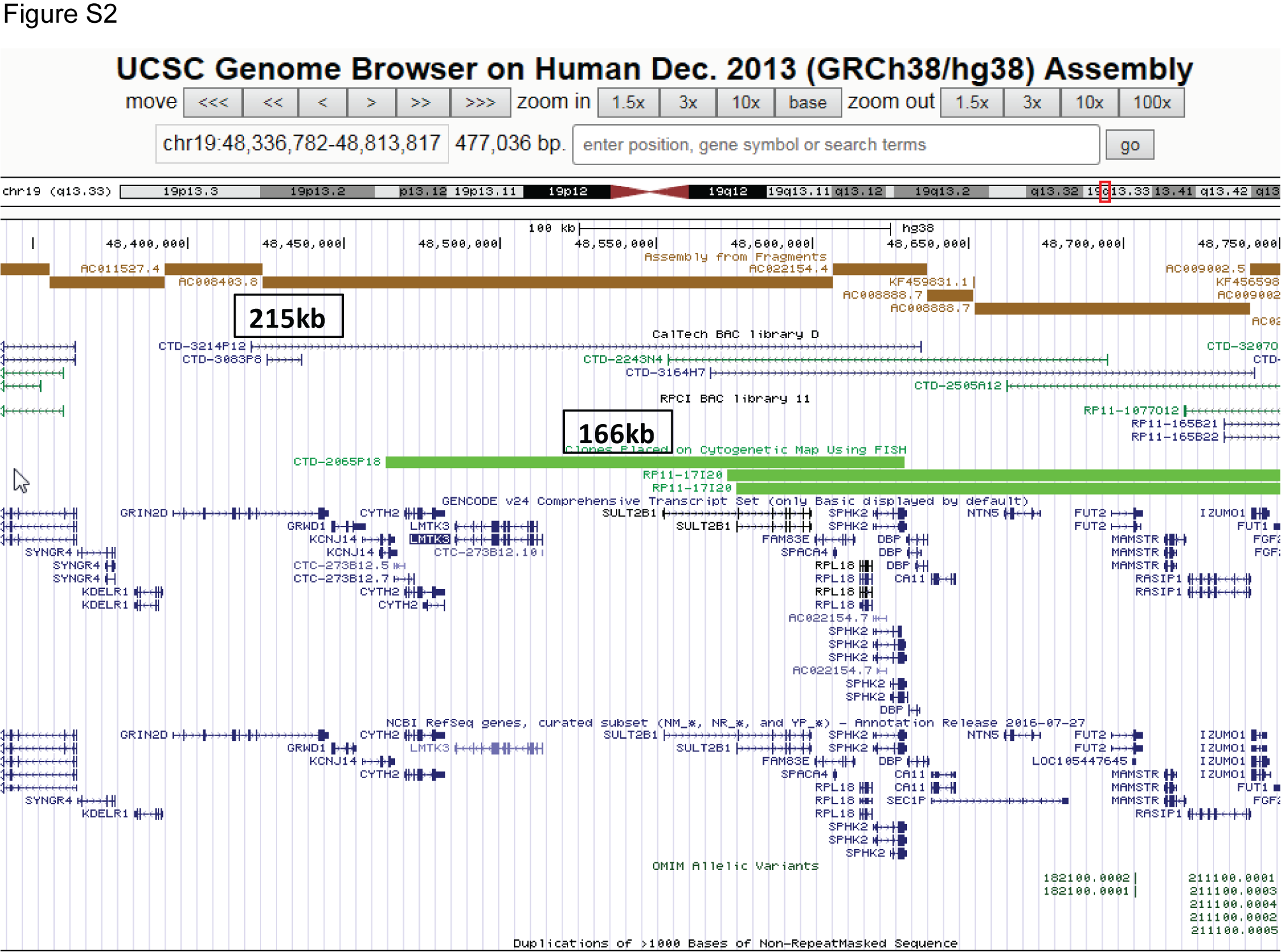

Supplement: Supplementary file 4 — Figure S2. LMTK3 DNA FISH probe map with two options for BACs that cover LMTK3 gene region which were 166 kb and 215 kb. (TIF 3156 kb) [file 13058_2019_1130_MOESM4_ESM.tif]

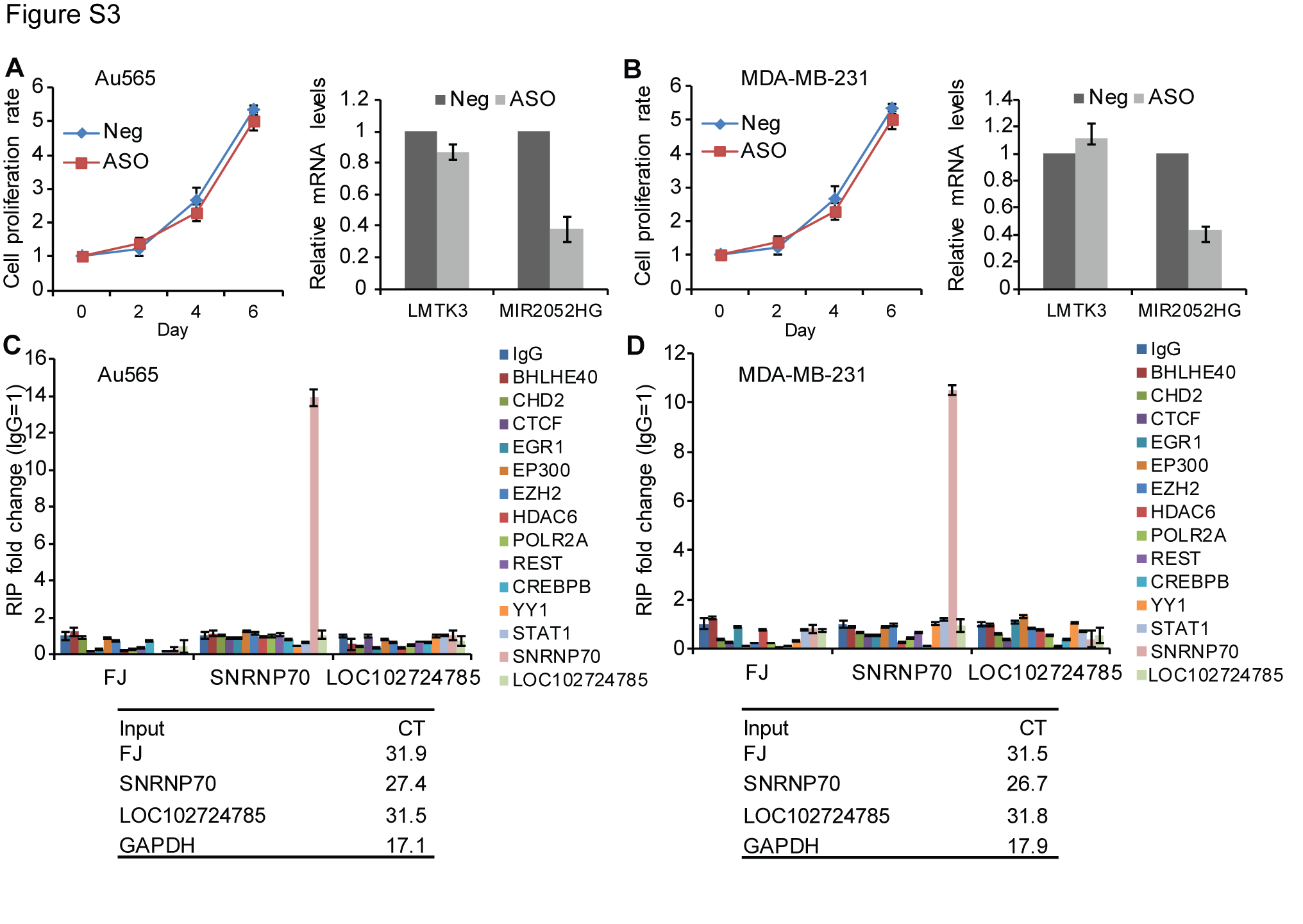

Supplement: Supplementary file 5 — Figure S3. Knockdown of MIR2052HG does not affect LMTK3 expression and proliferation of HER2+ and TNBC cells. a–b Cell proliferation of HER+ Au565 (a) and TNBC MDA-MB-231 (b) cells after knocking down MIR2052HG. LMTK3 gene expression and MIR2052HG knockdown efficiency was determined by qRT-PCR. c–d EGR1 antibody failed to immunoprecipitate MIR2052HG in Au565 (c) and MDA-MB-231 (d) cells. Error bars represent SEM of two independent experiments in triplicate. (TIF 1019 kb) [file 13058_2019_1130_MOESM5_ESM.tif]

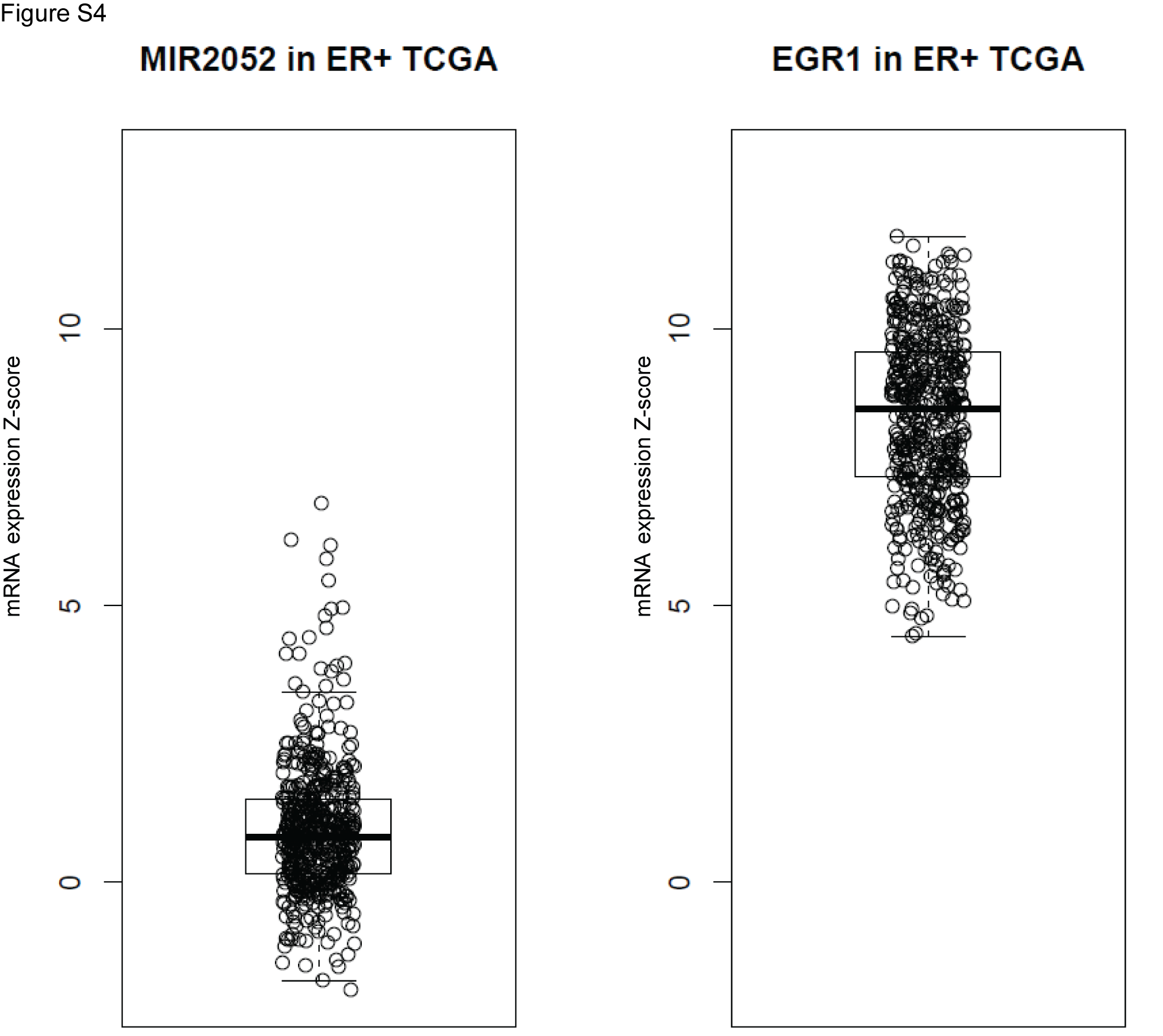

Supplement: Supplementary file 6 — Figure S4. MIR2052HG and EGR1 expression in TCGA ER-positive breast cancer patients. (TIF 1311 kb) [file 13058_2019_1130_MOESM6_ESM.tif]

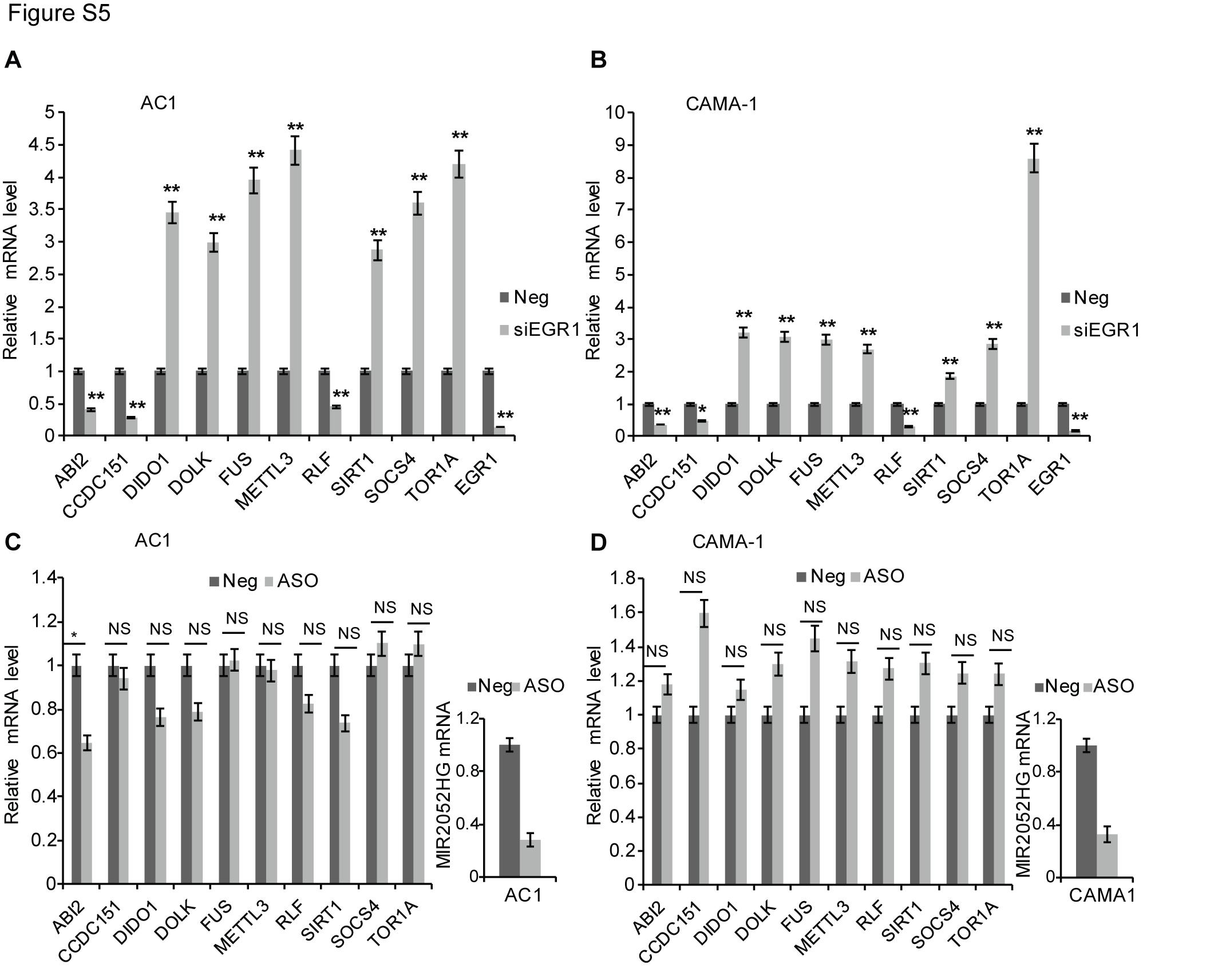

Supplement: Supplementary file 7 — Figure S5. Knockdown of MIR2052HG specifically reduces binding of EGR1 to the LMTK3 promoter, but not the other EGR1 targets. a–b Relative mRNA expression of EGR1 targeted genes after knockdown of EGR1 in MCF7/AC1 (a) and CAMA-1 (b) cells. Error bars represent SEM; *p < 0.05, **p < 0.01. c–d Relative mRNA expression of EGR1 targeted genes after knockdown of MIR2052HG in MCF7/AC1 (c) and CAMA-1 (d) cells. Error bars represent SEM; *p < 0.05, Non-significant (NS): p > 0.05. (TIF 1454 kb) [file 13058_2019_1130_MOESM7_ESM.tif]

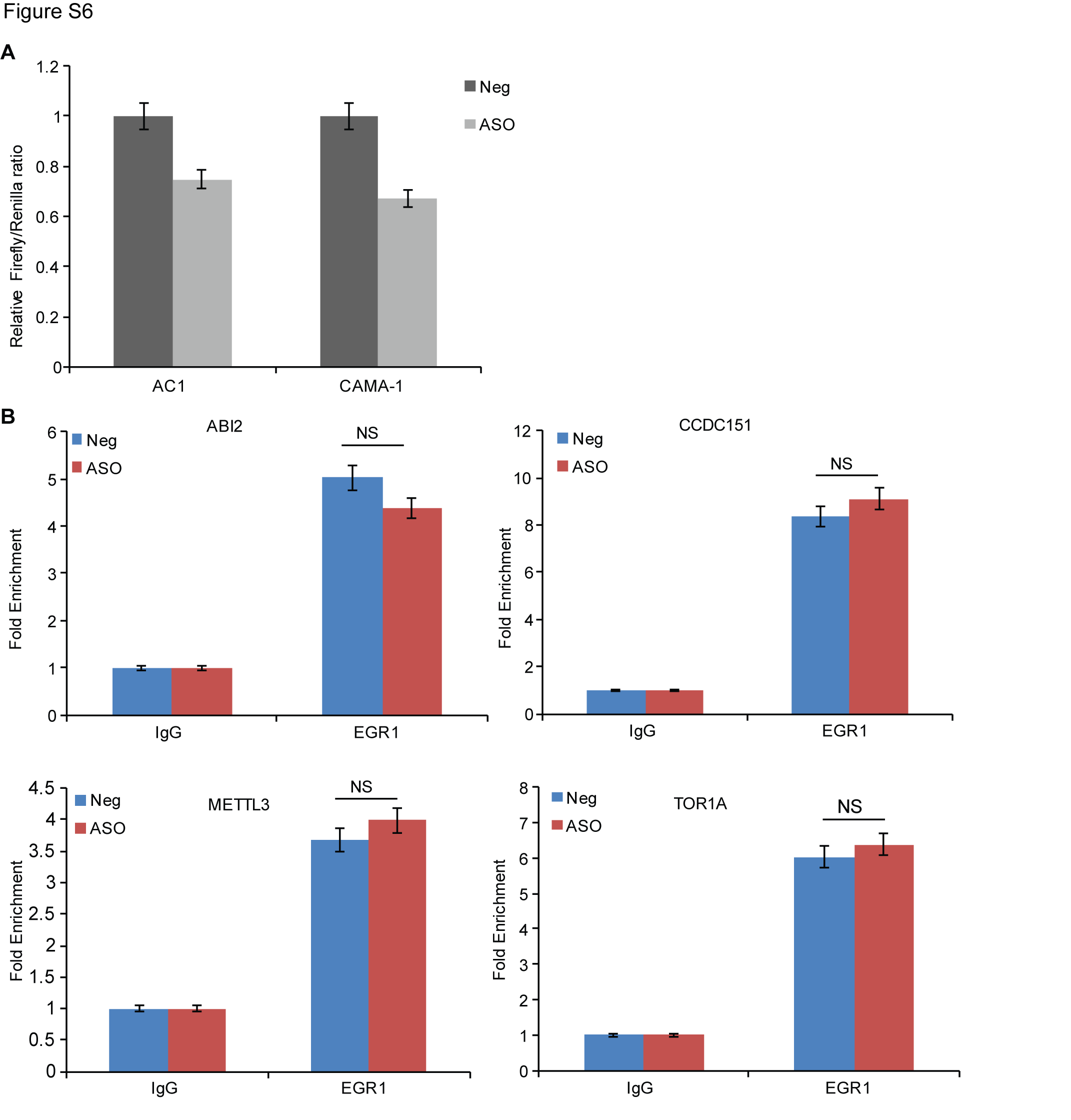

Supplement: Supplementary file 8 — Figure S6. MIR2052HG has no significant effect on other EGR1 targeted genes. a EGR1 reporter assay in MIR2052HG knocked-down MCF7/AC1 and CAMA1 cells. b ChIP analysis demonstrates binding of EGR1 to additional EGR1 targeted genes and knockdown of MIR2052HG has no impact on the binding. IgG serves as a control. Error bars represent SEM; Non-significant (NS): p > 0.05. (TIF 848 kb) [file 13058_2019_1130_MOESM8_ESM.tif]

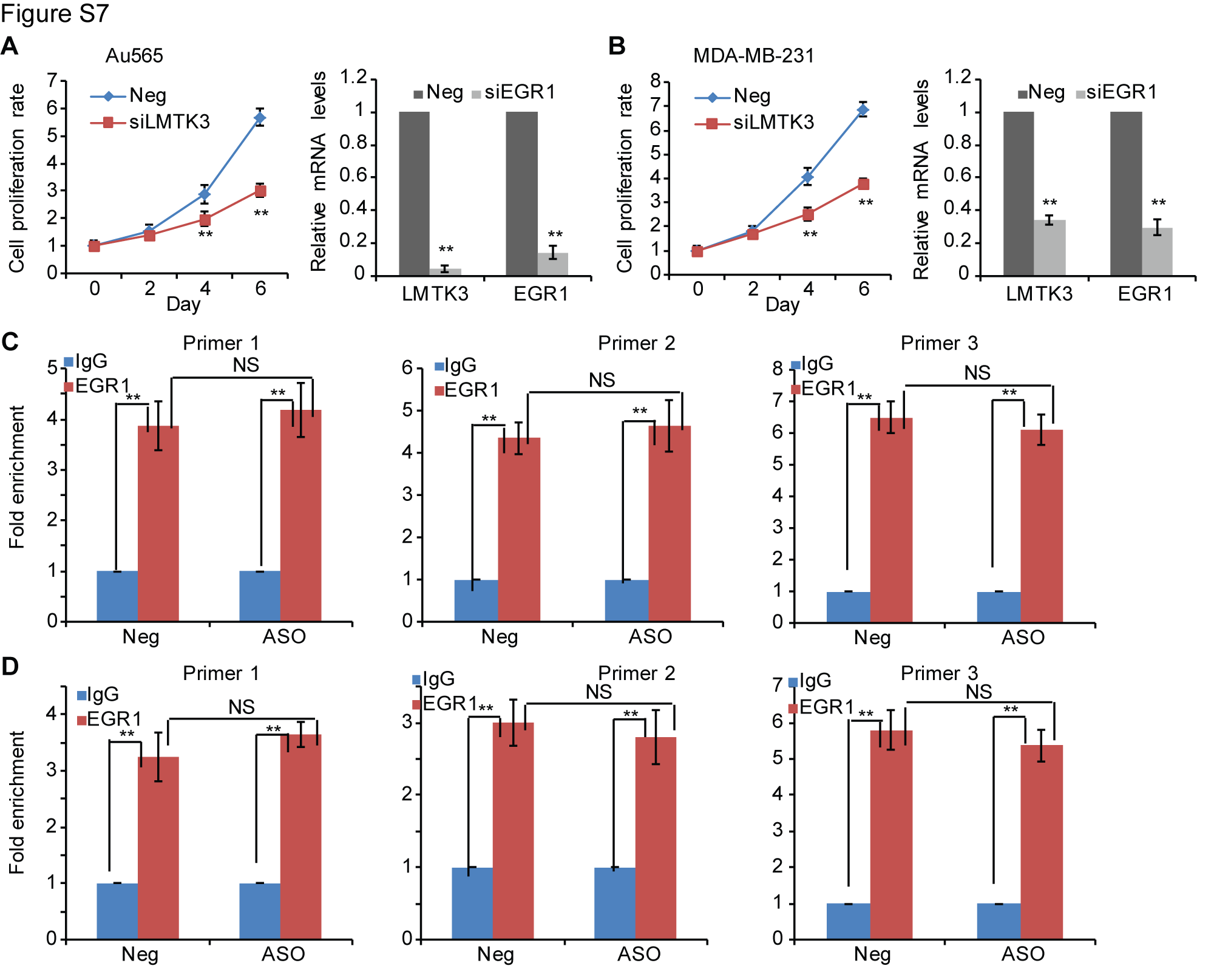

Supplement: Supplementary file 9 — Figure S7. EGR1, but not MIR2052HG, regulates LMTK3 expression in HER2+ and TNBC cells. a–b Cell proliferation of HER+ Au565 (a) and TNBC MDA-MB-231 (b) cells after knocking down LMTK3. LMTK3 gene expression and EGR1 knockdown efficiency was determined by qRT-PCR. c–d ChIP analysis demonstrates the binding of EGR1 to the promoter region of the LMTK3 gene locus in AU565 (c) and MDA-MB-231 (d) cells. However, knockdown of MIR2052HG did not change the binding. IgG serves as a control. Error bars represent SEM of three independent experiments in triplicate; **p < 0.01, Non-significant (NS): p > 0.05. (TIF 1117 kb) [file 13058_2019_1130_MOESM9_ESM.tif]

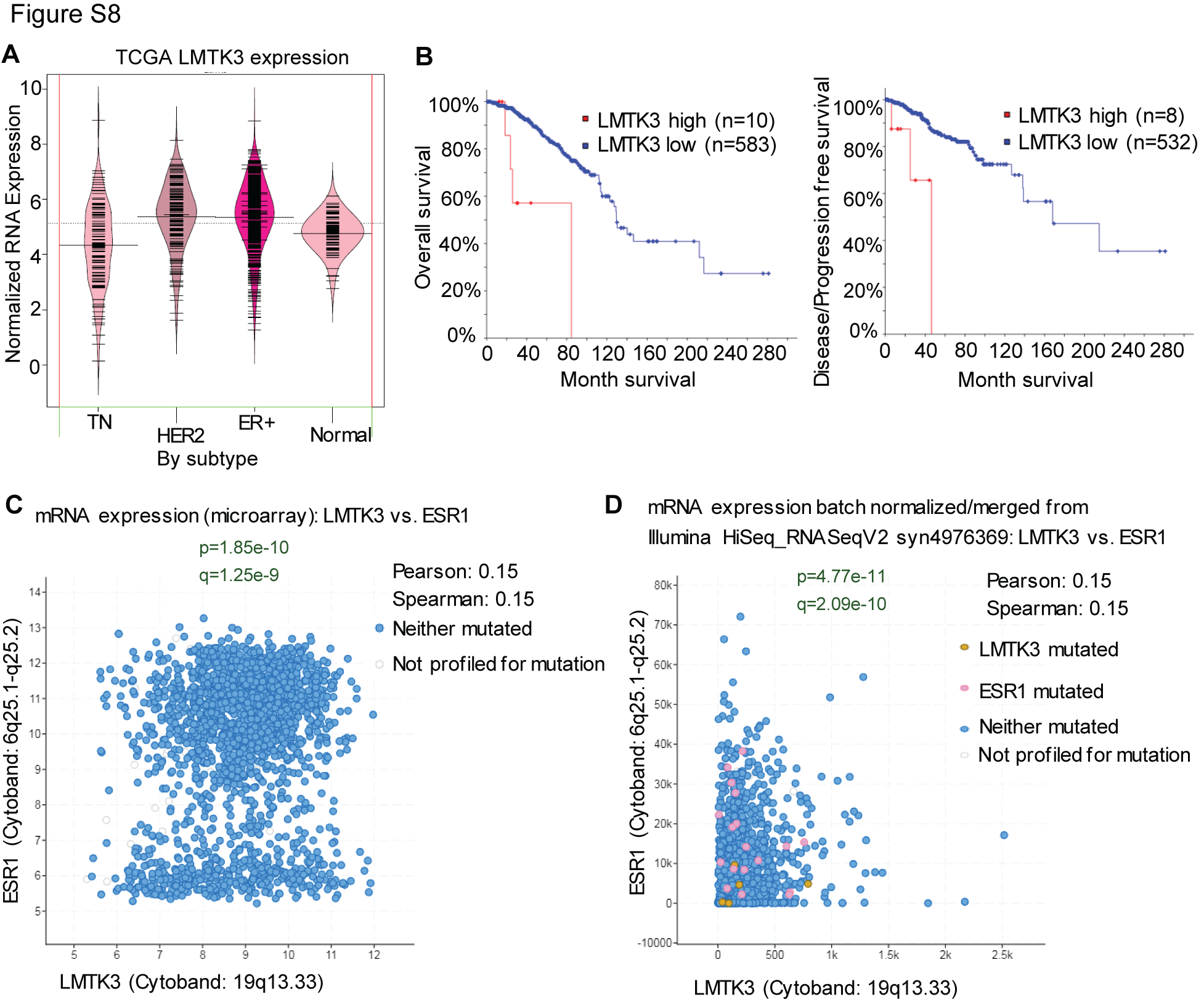

Supplement: Supplementary file 10 — Figure S8. Correlations of LMTK3 expression with ESR1. a LMTK3 expression in TCGA breast cancer patients. There are significant differences in the mean expression of LMTK3 among the four groups (HER2, ER+, TN, and Normal) using Kruskal-Wallis test (p < 2.2e−16). Pairwise comparison Wilcoxon test was also performed to determine the LMTK3 expression difference between the subtypes. Using Bonferroni correction for multiple testing, pairwise comparison showed: LMTK3 level in TN subtype is different from HER2 (p = 2.4e−08) and ER+ (p = 3.0e−11) but not significantly different from Normal (p = 0.17); LMTK3 in HER2 subtype is different from Normal (p = 1.5e−07) but not from ER positive (p = 0.892); while ER+ is significantly different from Normal (p = 6.5e−09). b Kaplan-Meier plots demonstrated the associations between LMTK3 expression level and overall survival (p = 3.927e−5) as well as disease-free survival (p = 9.587e−5) in TCGA ER-positive breast cancer patients. c Correlations of LMTK3 expression with ESR1 in 2509 METABRIC breast cancer patients. d Correlations of LMTK3 expression with ESR1 in TCGA breast cancer patients. (TIF 2271 kb) [file 13058_2019_1130_MOESM10_ESM.tif]
